# Molecular and Structural Characterization of Five Novel *GLA* Gene Variants in Fabry Disease

**DOI:** 10.3390/ijms27198669

**Published:** 2026-09-28

**Authors:** Monia Anania, Veronica Pagliardini, Marco Spada, Laura Econimo, Irene Giacalone, Martina Vinci, Paolo Colomba, Francesca Cappelletti, Carmelo Tonelli, Michelangelo Mancuso, Giulia Duro, Emanuela Maria Marsana, Marta Moschetti, Giovanni Duro

**Affiliations:** 1 Institute for Biomedical Research and Innovation (IRIB), National Research Council (CNR), 90146 Palermo, Italy; monia.anania@irib.cnr.it (M.A.); irene.giacalone@irib.cnr.it (I.G.); martina.vinci@irib.cnr.it (M.V.); paolo.colomba@irib.cnr.it (P.C.); emanuelamaria.marsana@irib.cnr.it (E.M.M.); marta.moschetti@irib.cnr.it (M.M.); 2 Department of Pediatrics, University of Torino, 10126 Torino, Italy; vpagliardini@gmail.com (V.P.); marco.spada@unito.it (M.S.); 3 Nephrology Unit, Department of Medical and Surgical Specialties, Radiological Sciences, and Public Health, University of Brescia—ASST Spedali Civili of Brescia, 25100 Brescia, Italy; 4 Nephrology Unit, Policlinico Le Scotte, 53100 Siena, Italy; f.cappelletti@ao-siena.toscana.it; 5 Cardiology Unit, Vittorio Emanuele Hospital, 93012 Gela, Italy; carmelotonelli@gmail.com; 6 Department of Clinical and Experimental Medicine, Neurological Institute, University of Pisa, 56126 Pisa, Italy; michelangelo.mancuso@unipi.it; 7 Department of Medical, Surgery and Health Sciences, University of Trieste, 34149 Trieste, Italy; giulia.duro@asugi.sanita.fvg.it; 8 Clinical Medicine and Rheumatology Unit, Cattinara University Hospital, 34149 Trieste, Italy

**Keywords:** Fabry disease, *GLA* gene, α-galactosidase A, Lyso-Gb3, novel mutations

## Abstract

Fabry disease is an X-linked lysosomal storage disorder caused by pathogenic variants in the *GLA* gene, encoding α-galactosidase A. Enzyme deficiency leads to progressive globotriaosylceramide (Gb3) accumulation and multisystemic involvement. Here, we characterize five previously undescribed *GLA* variants (p.D109N, p.N215T, p.N192H, p.L166P, and p.F248S) through an integrated approach combining biomolecular and computational analyses to investigate their effects on enzyme structure, catalytic activity, and dimerization. The identified substitutions affect residues located in regions critical for protein folding and active-site integrity. The p.F248S variant may destabilize the hydrophobic core and reduce thermodynamic stability, whereas p.D109N and p.N192H may disrupt hydrogen-bond networks required for proper catalytic geometry. The p.N215T substitution is associated with impaired glycosylation, while p.L166P may induce local conformational changes that compromise correct folding. Biochemical analyses of all samples carrying these variants revealed reduced α-galactosidase A activity and increased the Gb3 levels, but also of uncertain significance (VUS). Overall, these findings support an effect of the five variants on α-galactosidase A structure and function and highlight the value of integrating clinical, genetic, biochemical, and computational data for variant interpretation. Molecular characterization of novel variants may improve genetic diagnosis, inform therapeutic decisions, and support the development of targeted treatment strategies in Fabry disease.

## 1. Introduction

Fabry disease (FD, OMIM #301500) is a rare genetic lysosomal storage disorder, with an incidence of 1 in 80,000 live births [1]. It is a multisystemic disorder characterized by a progressive accumulation of glycosphingolipids, primarily globotriaosylceramide (Gb3), which accumulate in the lysosomes of numerous cell types, particularly in vascular endothelial cells [2,3,4,5]. Cellular accumulation is caused by the partial or total loss of function of the enzyme α-galactosidase A (α-GAL A) [6], resulting from mutations in the *GLA* gene, which is located on the long arm of the X chromosome (Xq22.1) [7]. The coding region of the *GLA* gene consists of 1290 base pairs (bp) and is divided into 7 exons (92–291 bp, Figure 1); it encodes the α-GAL A enzyme, comprising 429 amino acids, including the 31 amino acids of the N-terminal signal peptide [8].

The enzyme α-Gal A is a glycoprotein synthesised as a 50 kDa precursor, which is converted into the mature 46 kDa lysosomal form after removal of the signal peptide during transport to the lysosomes [9]. The X-ray crystallographic structure shows that α-GAL A is a homodimeric glycoprotein, with each monomer comprising two domains: a (β/α)_8_ domain containing the active site and a C-terminal domain formed by eight antiparallel β-strands arranged in two β-sheets in a β-sandwich structure (Figure 2) [10].

Each monomer contains five disulphide bonds and four N-glycosylation sites. The active enzyme is an acidic lysosomal hydrolase that needs saposin B to work [11]. Domain 1, which contains the active site, has a higher frequency of disease-causing point mutations, with 43% of the residues affected, whereas 28% of the residues in Domain 2 were found to be mutated in Fabry patients. Some mutations can destroy the enzyme’s active site by altering the residues that form it or that are essential for its correct three-dimensional structure; others may also alter residues located outside the active site, but which negatively affect the protein’s folding and stability; other mutations, on the other hand, cause a premature stop codon, resulting in a truncated and non-functional protein [12]. These changes lead to the enzyme not functioning correctly, resulting in the onset of the classic symptoms seen in Fabry patients. The clinical manifestations of the disease are progressive, with variation in onset, severity, age, and clinical course [13]. The classic clinical presentation of Fabry disease usually appears during childhood or adolescence, with angiokeratomas (particularly in the ‘swimsuit’ area), corneal opacities, microalbuminuria or proteinuria, and certain disorders of the autonomic nervous system. As patients age, the disease leads to significant deterioration of the renal and cardiovascular systems. The accumulation of glycosphingolipids in kidney cells impairs their function, leading to progressive nephropathy, which is one of the most serious features of Fabry disease [3]. In males with the classic form, α-GAL A activity is very low or absent: a diagnosis can be reliably made by measuring the enzyme’s activity in dried blood spots (DBFP analysis), plasma or isolated leukocytes; females, who are usually heterozygous and whose organs are chimeras of normal and diseased cells due to X-chromosome lyonization, generally exhibit less severe symptoms that are more difficult to detect [14]. Due to lyonization, females may have extremely variable α-GAL A activity; this makes it a less reliable first diagnostic step, as the levels can range between normal and pathological values even in the presence of causative mutations. The differences observed in enzyme activity between males and females were also observed in the accumulation of Lyso-Gb3: consequently, the Lyso-Gb3 test is also not reliable in females. In these individuals, genetic testing is the only accurate way to diagnose Fabry disease [15].

This wide range of clinical presentation in both male and female patients could be explained by genetic variability and the numerous mutations described in the literature (around 1000), as well as by the unclear role attributed to intronic mutations and SNPs (single nucleotide polymorphisms) [8]. Phenotypic heterogeneity manifests mainly in two forms of the disease: the classic form and the late-onset form [16]. The low correlation between genotype and phenotype is also reflected at the intra-family level, further contributing to the wide variety of symptoms, which often overlap with those of other diseases [17]. Future studies could help us understand the reasons behind this variability; one of the factors is likely to be epigenetics. In Fabry disease, the identification of new mutations is therefore important in order to link them to new phenotypes.

In this study, we describe five new mutations identified in the *GLA* gene of patients with a clinical diagnosis consistent with FD, using a biomolecular approach, and investigate the role that these mutations may play in the enzyme’s catalytic and dimerization activity through computational analysis. The identification of new mutations increases molecular understanding of the *GLA* gene and provides physicians with valuable support in making an accurate diagnosis of the disease. Furthermore, genetic testing of relatives of affected patients also allows us to identify pre-symptomatic individuals who can begin treatment as soon as possible, before organ damage becomes irreversible.

## 2. Results

In this study, we describe five new mutations (p.D109N, p.N215T, p.N192H, p.L166P and p.F248S) that we have identified in patients with clinically suspected Fabry disease. We carried out a comprehensive diagnostic process that included an evaluation of α-galactosidase A enzyme activity, genetic analysis of the *GLA* gene, and measurement of Lyso-Gb3 in the blood. The genetic alterations that we identified in these patients are not listed in the http://www.hgmd.org (accessed on 27 January 2026) and in the Fabry’s disease databases. We have designated the five mutations in accordance with the guidelines for mutation naming recommended by the Human Genome Variation Society (www.HGVS.org/varnomen, accessed on 27 January 2026). We therefore proceeded with a computational analysis of the possible effect that the substitutions might have on the structure and function of the protein, using the Polyphen 2 tool (http://genetics.bwh.harvard.edu/pph2/, accessed on 27 January 2026), also considering the conservation of amino acids in different species: PolyPhen-2 predicts the effect of a missense variant on protein function, assigning a score between 0 and 1: if the score is close to 0, the result is interpreted as indicating a higher probability of a benign effect; if it is close to 1, a higher probability of a deleterious effect. Using the DynaMut2 software (https://biosig.lab.uq.edu.au/dynamut2/, accessed on 3 March 2026), we obtained detailed information about the protein sequences associated with three-dimensional structures and the protein stability, which is one of the possible mechanisms through which a variant can alter the function of the protein. Using the Fabry_CEP web application (https://web.na.icb.cnr.it/fabry_cep/, accessed on 15 March 2026, last update on 21 October 2016), we examined the eligibility of patients for treatment with pharmacological chaperones.

### 2.1. CASE 1: p.D109N MUTATION

This case was referred to us by the paediatric department, as it concerned a 5-month-old baby. Given the patient’s young age (5 months), the clinical symptoms do not provide clear evidence of Fabry disease and the possibility of a presymptomatic stage, and further follow-up will be necessary.

We therefore initially tested the sample using a biochemical analysis of enzymatic activity, which was found to be 0.5 nmol/mL/h, well below the reference values, and the Lyso-Gb3 concentration was found to be 1.78 nmol/L (Table 1).

In light of the pathogenic nature of the biochemical results, we therefore proceeded to analyze the seven exons of the *GLA* gene and the intronic regions flanking the exons. In exon 2 of the gene, we identified, in hemizygous, the new missense mutation c.325 G>A, which causes the substitution of a guanine for an adenine at position 325 of the cDNA (Figure 3), resulting in the substitution of an aspartic acid for an asparagine at position 109 of the protein (p.D109N).

The substitution of aspartic acid with asparagine at position 109 of the A chain of α-galactosidase A may alter the structure of the protein, compromising its function, and could therefore be the cause of Fabry disease: indeed, the sample under examination exhibited enzymatic activity close to 0. According to Polyphen 2 (http://genetics.bwh.harvard.edu/pph2/, accessed on 27 January 2026), a tool that predicts the potential effect of an amino acid substitution on the structure and function of a human protein, we can predict that D109 is highly conserved in evolution, and that the mutation could be deleterious, with a score of 1. The combination of clinical, biochemical, and predictive data supports the idea that the p.D109N mutation may play a key role in Fabry disease (Figure 4).

Another bioinformatic tool we used was DynaMut2 (https://biosig.lab.uq.edu.au/dynamut2/, accessed on 3 March 2026), which allowed us to make a quantitative prediction of the effect of the p. D109N amino acid substitution on the stability of the protein in question. The results showed a change in Gibbs free energy (ΔΔG), an indicator of stability, of −1.35 kcal/mol, indicating that the mutation has a highly destabilising effect on the protein’s structure. The negative value of ΔΔG suggests that the substitution of the amino acid aspartic acid with asparagine at position 109 reduces the conformational stability of the domain examined compared with the wild type, and is likely to affect the function of the protein itself (Figure 5).

Furthermore, in the Fabry_CEP database (https://web.na.icb.cnr.it/fabry_cep/, accessed on 15 March 2026), this variant has not been reported in the literature; the substitution is located within the (β/α)_8_ TIM-barrel domain but does not directly involve the active site and does not disrupt disulphide bonds, and has an 87 per cent probability of responding to pharmacological chaperones (Figure 6).

### 2.2. CASE 2: p.N215T MUTATION

The proband is a 56-year-old man who came to our attention with a typical clinical presentation, as he presented with episodes of proteinuria and left ventricular hypertrophy (LVH).

Clinical suspicion led us to carry out molecular analysis in this patient, beginning with an assessment of enzymatic activity which, as shown in Table 2, was found to be almost zero in the patient (0.8 nmol/mL/h), with a high level of LysoGb3 accumulation (21.38 nmol/L) (Table 2).

Genetic analysis of the *GLA* gene revealed a heterozygous substitution of an adenine for a cytosine at position 644 of the cDNA, which results in the substitution of an asparagine for a threonine at position 215 of the protein (Figure 7).

The c.644 A>C mutation has not previously been described in the literature and is not reported in databases of mutations associated with Fabry disease: a different mutation, c.644 A>G, p.N215S, which occurs at the same site, has been found in patients with an atypical form of Fabry disease [18].

The presence of clinical signs characteristic of Fabry disease encouraged us to investigate, using the Polyphen-2 platform, the mutation that was found to be possibly damaging with a score of 0.49, partly due to the conservation of the amino acid asparagine at position 215 in the evolutionary tree (Figure 8). A score of 0.49 indicates a condition that is potentially dangerous, supporting the hypothesis that it may be pathological.

The asparagine at position 215 is located within the N-terminal catalytic domain of the enzyme and represents an important glycosylation site for lysosomal transport and enzymatic stability [19]. Therefore, similarly to the well-known p.N215S mutation, the p.N215T mutation also causes the loss of the glycosylation site; however, given that both serine and threonine are polar, hydrophilic amino acids and can form hydrogen bonds, the only reason for the different behaviour of the two mutations (p.N215T being the classic form and p.N215S the atypical form) is that threonine has an extra methyl group, leading to greater steric interference and affecting the efficiency of α-GalA.

According to the Dynamut-2 tool, the ΔΔG for this mutation is around 0.22 kcal/mol, a value considered to be of little significance in terms of stability. A mutation with a ΔΔG of 0.22 kcal/mol does not suggest a serious folding defect and does not indicate a significant destabilization of the overall structure. However, as asparagine at position 215 is an important N-glycosylation site, its mutation to threonine results in the loss of the glycosylation site, causing a loss of function, and this is far more significant than the ΔΔG (Figure 9).

In the Fabry_CEP database, the p.N215T variant has not been reported in the literature, the mutation does not occur in the active site and does not disrupt disulphide bonds; it also has a PSSM score (a measure of the extent to which a mutation ‘breaks’ the evolutionary conservation of the protein) of −1, thus indicating that the mutation is more likely to be pathogenic (Figure 10).

### 2.3. CASE 3: p.N192H MUTATION

The proband is a 56-year-old man who is being investigated for the characteristic clinical signs of Fabry disease, such as left ventricular hypertrophy (LVH) and renal failure requiring dialysis.

The patient’s biomolecular analysis revealed a very low enzyme activity level (1.1 nmol/mL/h), with a LysoGb3 substrate accumulation level well above the threshold value (29.14 nmol/L) (Table 3).

The subsequent genetic analysis of the *GLA* gene allowed us to identify a new hemizygous mutation within exon 4, which causes the substitution of an adenine for a cytosine at position 574 of the cDNA, resulting in the substitution of an asparagine for a histidine in codon 192 of the protein (Figure 11a).

We extended the investigation to the proband’s daughter, a 26-year-old woman, who, in addition to carrying the same heterozygous mutation as her father (Figure 11b), had enzyme activity that was absolutely normal (9.5 nmol/mL/h, a value consistent with the phenomenon of lyonization) but a pathological substrate accumulation value (4.45 nmol/L).

The presence of an abnormal enzymatic activity level in the proband, combined with an accumulation level above the normal range in both subjects studied in this case, suggests that this mutation plays a pathogenic role. This hypothesis is supported by the use of Polyphen 2, which classifies this mutation as a variant that could adversely affect the protein’s function, assigning it a score of 1, thus supporting the hypothesis that the mutation may be highly deleterious, not least because of the conservation of asparagine at position 192 across the evolutionary tree (Figure 12).

Asparagine at position 192 also falls within the N-terminal catalytic domain: it is therefore located near the catalytic site but is not one of the amino acid residues directly involved in catalysis (such as aspartic acid at positions 170 and 231). The replacement of asparagine with histidine could alter the stability of the TIM barrel domain, the folding of the active site, and consequently, possibly the affinity for the substrate.

The results predicted by the DynaMut2 tool showed a change in Gibbs free energy (ΔΔG) associated with the p.N192H mutation of −0.96 kcal/mol, indicating that the mutation has a highly destabilising effect on the protein’s structure. The negative value of ΔΔG suggests that the substitution of the amino acid asparagine with histidine at position 192 reduces conformational stability, which is likely to affect the function of the protein itself (Figure 13).

In the Fabry_CEP database, the p.N192H mutation has not been reported in the literature; it does not occur directly within the active site but close to it, and does not break any disulphide bonds; furthermore, it has a PSSM score of −2, indicating a high probability that the mutation is pathogenic. It also has a 59 per cent probability of responding to pharmacological chaperones (Figure 14).

### 2.4. CASE 4: p.L166P MUTATION

The patient is a 57-year-old woman who presented with left ventricular hypertrophy typical of Fabry disease. Clinical suspicion encouraged us to proceed with genetic analysis of the *GLA* gene, which revealed a heterozygous variant in exon 3, involving the substitution of a thymine for a cytosine at position 497 of the cDNA, resulting in the substitution of a leucine for a proline at position 166 of the protein (Figure 15).

The test carried out on the DBS showed an enzymatic activity of 3.5 nmol/mL/h (consistent with lyonization (3), and an accumulation value above the threshold values (17.05 nmol/L) (Table 4).

The mutation described is not listed in the databases of mutations associated with Fabry disease and has not previously been reported in the literature; however, the presence of LysoGb3 accumulation in the blood observed in this patient suggests that it plays a pathogenic role. When using Polyphen 2 to predict the severity of the mutation, this variant was assessed as being capable of negatively affecting the protein’s function. A score of 1 indicates that the mutation is considered deleterious, supporting the hypothesis that it may be pathogenic and indicating the conservation of the amino acid leucine at position 166 along the evolutionary tree (Figure 16).

Leucine at position 166 is located within the N-terminal catalytic domain, contributing to the formation of the edge of the enzyme’s active site within which the catalytic reaction takes place. Structural analyses of closely related mutations (such as p.L166V or other substitutions at this position) show that residues in this region cover the edge of the active site pocket and contribute to the correct orientation of the substrate within the catalytic centre [15,18,20,21]. Proline, on the other hand, has some distinctive characteristics as an amino acid: it has a rigid ring that disrupts secondary structures, such as α-helices or β-sheets, and influences the local conformation of the peptide chain. This may result in alterations in the folding of the local chain and/or in its interaction with the substrate, the enzyme’s inability to form the active site correctly and thus to position the substrate for catalysis, and impaired or unstable dimerization [22].

If the p.L166P mutation alters the structure of the active site or the stability of the catalytic domain, it is highly likely that the enzymatic activity of α-galactosidase A will be reduced or completely lost. This can lead to the accumulation of Lyso-Gb3 in the lysosomes, as the enzyme fails to break down the substrate correctly, resulting in progressive multisystemic cellular and tissue damage characteristic of the disease.

These data are also supported by a change in Gibbs free energy (ΔΔG), predicted using the DynaMut-2 tool, which is highly negative at −1.87 kcal/mol. This indicates that the substitution of leucine at position 166 with proline significantly reduces the enzyme’s stability, thus decreasing its functionality (Figure 17).

In the Fabry_CEP database, the p.L166P mutation has not been reported in the literature; it does not occur directly within the active site, and therefore likely alters the stability of the catalytic domain; nor does it break any disulphide bonds; it also has a PSSM score of −2, indicating that this mutation is likely to be highly pathogenic (Figure 18).

### 2.5. CASE 5: p.F248S MUTATION

The proband is a 48-year-old woman who presented with acroparesthesia, leukopathy, and neurological symptoms typical of Fabry disease, such as TIA or stroke. Furthermore, her father had died with a clinical picture with clinical symptoms consistent with Fabry disease, but not confirmed as the patient had died. According to the family history, the patient has two sisters, the older of whom has ABS and the younger of whom also has a history of TIA.

We therefore carried out a genetic analysis of the seven exons of the *GLA* gene and the IVS4 intron in the proband, which revealed the c.743 T>C mutation in exon 5 in a heterozygous state: this is a substitution of a thymine for a cytosine at position 743 of the cDNA, which results in the replacement of a phenylalanine with a serine in codon 248 of the protein (Figure 19). Finally, we performed the MLPA analysis with a negative result.

Subsequent analysis of the biochemical data revealed normal levels of α-galactosidase A enzymatic activity and normal levels of Lyso-Gb3 in the blood, consistent with lyonization (Table 5).

The mutation is not listed in the databases of mutations associated with Fabry disease and has not previously been described in the literature in patients with Fabry disease. According to Polyphen 2, we can predict that F248 is not highly conserved in evolution, and that the mutation would appear to be non-deleterious, with a score of 0.032. The combination of clinical, biochemical, and predictive data supports the view that the p.F248S mutation may not be the cause of the disease (Figure 20).

Residue 248 falls within the N-terminal catalytic domain of the enzyme. The phenylalanine at position 248 is a hydrophobic amino acid, which being located within the structural core, contributes to the three-dimensional stability of the domain itself.

The substitution of phenylalanine with serine at position 248 results in the removal of phenylalanine’s large hydrophobic side chain and the subsequent introduction of serine’s small, polar, and potentially reactive side chain [10,23]. This type of change is typical of mutations that disrupt the internal stability of the protein rather than directly altering its active site. Structural analyses of α-galactosidase A have shown that many mutations compromise the very core of the enzyme, affecting its folding and stability rather than its direct catalytic action itself [24]. Phenylalanine is less prone to alterations in patients with Fabry disease than other residues (such as tryptophan, cysteine, tyrosine, and glycine), with 5 out of the 15 phenylalanine residues (33%) affected by mutations that cause the disease: this is unexpected for a large hydrophobic residue typically found within a protein [8].

This finding is also supported by the positive change in Gibbs free energy, amounting to +0.44 kcal/mol, a value which indicates a very slight destabilization of the protein, likely with minimal structural impact, and which does not alter the enzyme’s functionality (Figure 21).

In the Fabry_CEP database, the p.F248S mutation has not been reported in the literature; it does not occur directly within the active site and therefore is likely to alter the stability of the catalytic domain and does not break any disulphide bonds; it also has a PSSM score of −2, indicating a likely high pathogenicity for this mutation (Figure 22), as this would be inconsistent with the biochemical and computational predictive results presented, as well as with the ACMG classification. We intend to investigate the role of this mutation further through subsequent family studies.

## 3. Discussion

Analysis of mutations in the *GLA* gene is a fundamental step in the diagnosis of Fabry disease in patients with a suspected clinical diagnosis. It is extremely important to diagnose affected patients so that the most appropriate treatment can be administered promptly. To date, more than 1000 mutations in the *GLA* gene responsible for Fabry disease have been described, including missense and nonsense mutations, small and large deletions and insertions, as well as gene rearrangements, including small and large intragenic deletions and insertions.

In this study, we describe five new missense mutations (p.D109N, p.N215T, p.N192H, p.L166P, and p.F248S) found in unrelated patients, confirming the heterogeneity observed in individuals with FD. Where possible, we have extended the genetic and enzymatic analysis to include relevant family members in order to represent and make a significant contribution to our understanding of the variants. The discovery of new genetic variants is essential for expanding the database of known mutations and for improving genetic diagnosis. Extending biomolecular and molecular diagnostic testing to family members is particularly important for identifying carriers or asymptomatic affected individuals, thus supporting preventive treatment and closer clinical monitoring.

To further our study of these new variants, we not only used important biomolecular assays such as enzyme activity and substrate accumulation, but we also employed artificial intelligence (AI) systems applied to biology, such as Polyphen-2, DynaMut2, and Fabry_CEP. The integrated use of these systems has led to an accurate prediction of how these mutations might alter the three-dimensional structure of the protein, and consequently, what role they might play in FD.

In the case of the c.325 G>A (p.D109N) mutation, this results in reduced activity of the enzyme α-galactosidase A, and bioinformatics tools suggest that the mutation may have a highly destabilizing effect on the protein’s structure, thereby affecting the protein’s function. The combination of clinical, biochemical, and predictive data supports the idea that this mutation may play a key role in FD. In the case of the c.644 A>C (p.N215T) mutation, the integrated approach used in this study allowed us to predict that this variant may negatively affect the protein’s function, leading us to hypothesise that it may be a potentially pathogenic mutation; furthermore, when comparing this new mutation with the well-known p.N215S mutation, both have a very strong common feature: they eliminate Asn215 and thus the N-glycosylation site; however, the substitution of Asn215 with serine results in a relatively small change, whereas threonine is a slightly bulkier residue, leading to a slightly greater steric effect. The c.574 A>C (p.N192H) mutation results in pathological enzyme activity in the proband, associated with an above-normal accumulation level in both subjects analysed; in conjunction with the bioinformatic data obtained, we can predict the negative role that this variant may play in FD, as it would adversely affect the protein’s function, having a highly destabilizing effect on the protein’s structure. The c.497 T>C (p.L166P) mutation results in the presence of Lyso-Gb3 in the blood at levels above the threshold value; this finding, combined with the information obtained from bioinformatics tools, allows us to predict that this variant significantly reduces the stability of the enzyme, thereby reducing protein function, and may play a negative role in FD. Furthermore, Leu166 is located in the central region of α-galactosidase A (α-Gal A), the lysosomal enzyme encoded by the *GLA* gene. An important point is that L166 is a residue already recognised as being susceptible to substitutions: in addition to L166V, other variations at this position have been described, including L166G. A structural/comparative study lists L166V amongst the variants associated with the Fabry phenotype and shows that the residue is located in a region where amino acid changes can impair the protein’s proper function. The last mutation, c.743 T>C (p. F248S), does not affect the biomolecular tests; therefore, according to a predictive analysis, the mutation would appear to be non-harmful and not a cause of FD.

This evidence, combined with clinical and biochemical data, has enabled us to classify the new variants described in this article in accordance with the ACMG classification criteria and guidelines [25]. The D109N mutation is located within the TIM-barrel catalytic domain (PM1) and is absent in the control sample (PM2). Biochemical studies have demonstrated a variation in residual enzymatic activity (PS3), and furthermore, multiple computational analyses—for example, Polyphen 2, UniProt, and AlphaFold—support the hypothesis of a deleterious effect on the gene or its product (PP3). In light of these pathogenicity criteria and given the presence of two moderate pieces of evidence and one strong piece of evidence supporting pathogenicity, as indicated in the ACMG Standards and Guidelines, this mutation can be considered ‘Likely Pathogenic’ (Table 6).

The N215T mutation meets the PM1, PM2, and PP3 pathogenicity criteria; it satisfies the PM5 pathogenicity criteria because variants at codon 215 that have already been described as pathogenic (e.g., N215S) exist, and this supports the interpretation that a different substitution at the same residue is pathogenic. Based on these pathogenicity criteria, and given that there are three indicators of moderate evidence of pathogenicity, the mutation can be classified as ‘Likely Pathogenic’ (Table 6).

The N192H mutation meets the PM1, PM2, and PP3 pathogenicity criteria; it also meets the supporting pathogenicity criteria PP1, since the two subjects may provide evidence of segregation/genotype (father–daughter). In accordance with these pathogenicity criteria, and given that there are two indicators of moderate evidence of pathogenicity and two supporting indicators, the variant can be classified as ‘Likely Pathogenic’ (Table 6).

The L166P mutation meets the pathogenicity criteria for classification as a ‘Likely Pathogenic’ variant, satisfying the PM1, PM2, PS3, and PP3 pathogenicity criteria. The mutation is, in fact, located within the enzyme’s catalytic domain; it was absent from the control sample, and the use of bioinformatics software supports the hypothesis that it has a deleterious effect on the gene or its product (Table 6).

The F248S mutation meets the PM2 pathogenicity criteria, but there is insufficient biochemical evidence to support its pathogenicity; therefore, as indicated in the ACMG Standards and Guidelines, this mutation may be considered of uncertain significance (VUS) (Table 6).

The fact that in most of the patients described in this article, the activity of the α-galactosidase A enzyme was normal is not surprising, as this is due to the location of the *GLA* gene on the X chromosome and the phenomenon of lyonization, that is, the inactivation of one of the two X chromosomes in the various cells of the female body. It has been observed that in some women, X chromosome inactivation is not random across all cells; instead, some of these cells exhibit a phenomenon of asymmetric inactivation [8]. This results in considerable clinical heterogeneity, ranging from the absence of symptoms to a clinical presentation as severe as that seen in males with Fabry disease [25,26,27]. This heterogeneity can also be observed in the levels of enzymatic activity, which is normal in over 70% of female patients, and of Lyso-Gb3 in the blood, which is normal in 80% of female patients with a late-onset phenotype and in 17% of women with a classic phenotype [15]. The α-Gal A and Lyso-Gb3 activity levels observed in the patients described in this article reflect these percentages.

All of the new mutations described in this article enhance our molecular understanding of the *GLA* gene and the heterogeneous nature of Fabry disease. Furthermore, the results of this study expand the mutational picture of the *GLA* gene and confirm the value of an integrated clinical, biochemical, and genetic approach to the diagnosis of Fabry disease.

## 4. Materials and Methods

### 4.1. Patients

Peripheral blood was collected, using EDTA as an anticoagulant, and dried on absorbent paper (dried blood spot, DBS). Genetic and enzymatic studies were performed at the Centre for Research and Diagnosis of Lysosomal Storage Disorders of IRIB-CNR in Palermo.

### 4.2. Genetic Analysis

Genomic DNA was isolated from the dried blood spot using silica-coated magnetic particles, in a robotic workstation for the automated purification of nucleic acids, EZ1 Purification Kits (Qiagen, Hilden, Germany). DNA concentrations were estimated using a biophotometer (Eppendorf, Hamburg, Germany). The search for mutations in the *GLA* gene was performed through Sanger sequencing. Eight pairs of primers were designed to analyze eight target regions containing the seven exons of the *GLA* gene, including the flanking regulatory sequences, and the cryptic exon. PCR products were purified and sequenced at Eurofins Genomics (Ebersberg, Germany).

We conducted an MLPA analysis on the DNA samples to identify major rearrangements of the *GLA*, genes, using the SALSA MLPA Kits P159-A5 *GLA*, in accordance with the manufacturer’s instructions (MRC Holland, Amsterdam, The Netherlands).

### 4.3. α-Galactosidase A Activity Assay

α-Galactosidase A activity assays were performed using the dried-blood filter paper (DBFP) test described by Chamoles et al. [28], with modifications [unpublished data]. A spot of 10 μL of blood on a circle of paper 6 mm in diameter was placed into a 96-well plate, suitable for fluorometric assays, and incubated for 18 h at 37 °C in a thermomixer; the reaction was terminated by the addition of 250 μL of 0.1 mol/L ethylenediamine (pH 11.4). The background fluorescence, i.e., fluorescence that is not due to specific enzyme activity, was determined for each sample, conducting another reaction in the presence of 0.14 mmol/L 1-deoxygalactonojirimycin (DGJ, the inhibitor of alpha galactosidase A) in citrate phosphate buffer (pH 4.5), instead of incubating the substrate separately, as in the Chamoles method. This background was subtracted from the fluorescence of the sample. In each assay, we added positive and negative controls and a calibration curve with 4-methylumbelliferone. Normal values were considered to be > 3.0 nmol/mL/h.

### 4.4. Lyso-Gb3 Determination

The determination of Lyso-Gb3 in blood was performed using the tandem mass spectrometry (LC-MS/MS) methodology as previously described by Polo et al. [29]; we sent the samples for the determination of lyso GB3 to Centogene via outsourcing (Rostock, Germany).

## Figures and Tables

**Figure 1:**
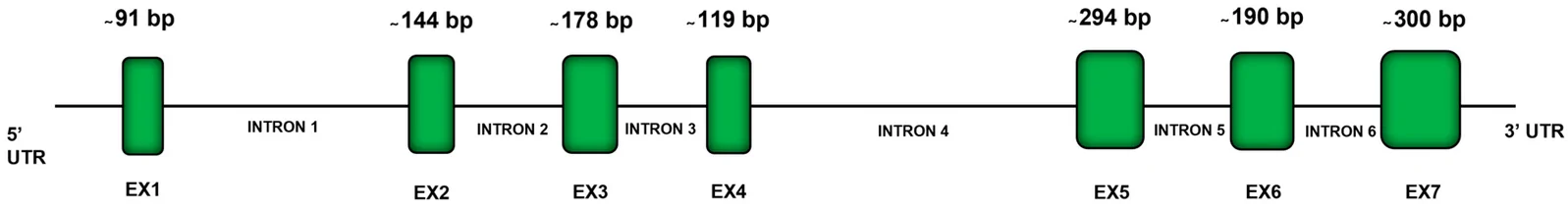
Diagram of the *GLA* gene.

**Figure 2:**
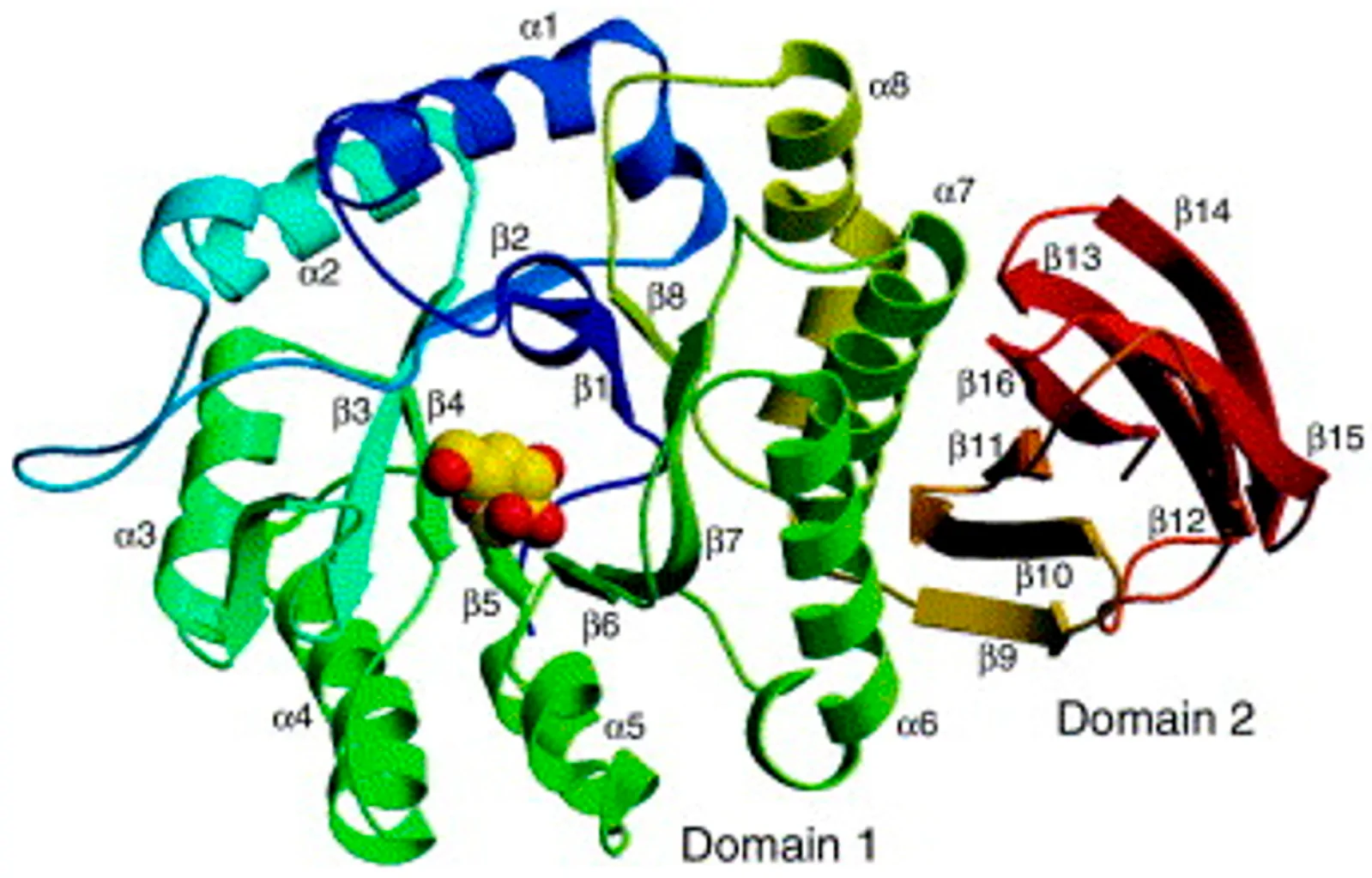
Crystallographic structure of α-GAL. Domain 1 contains the active site at the middle of the β-sheets within the (β/α)_8_ barrel, and Domain 2 consists of antiparallel β-sheets.

**Figure 3:**
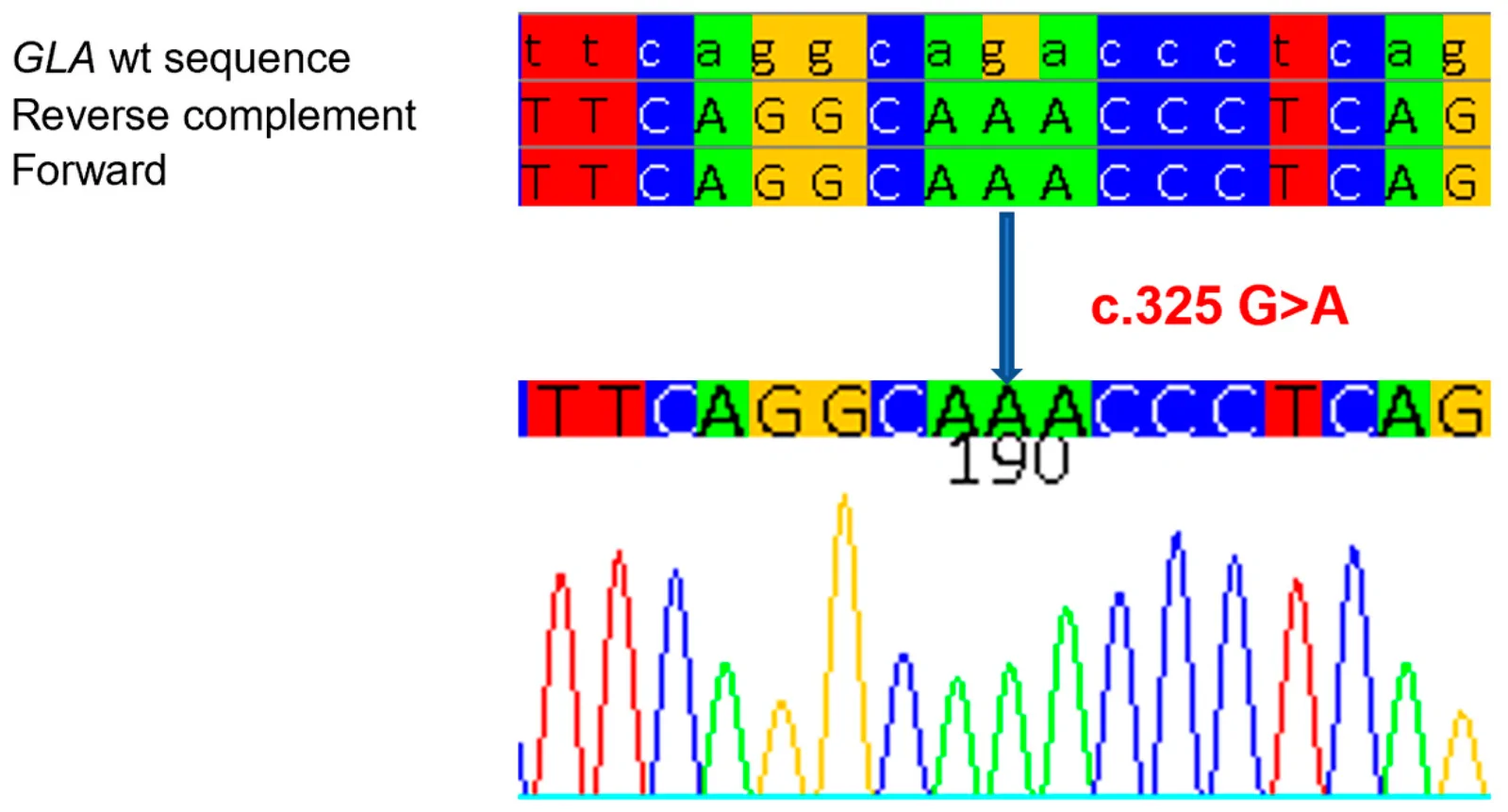
Sanger sequencing shows the c.325 G>A mutation in hemizygous state in the proband. Each base corresponds to a color: red for Thymine, blue for Cytosine, yellow for Guanine, green for Adenine. Arrows indicate the mutation.

**Figure 4:**
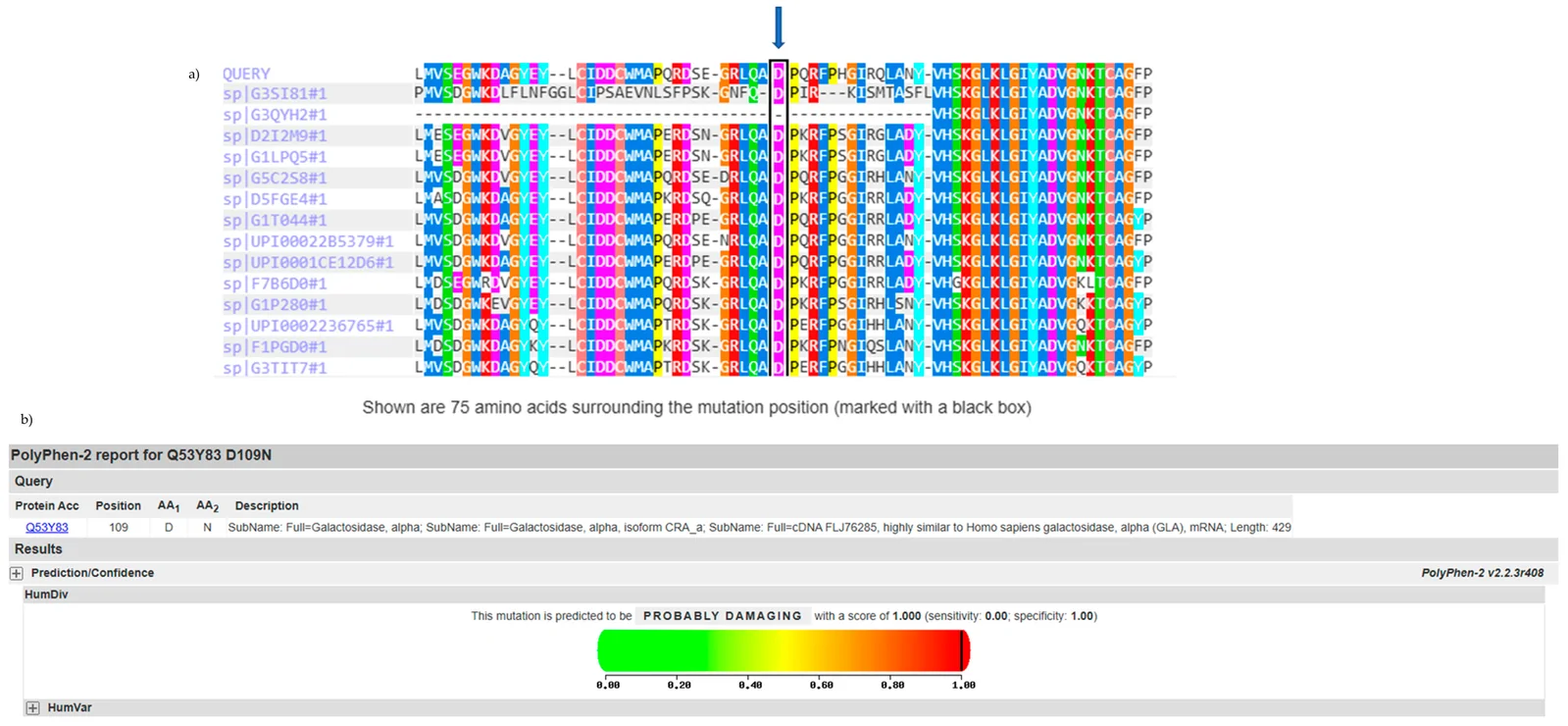
(**a**) Alignment of human α-Gal A amino acids with sequences from other organisms. The arrow indicates the amino acid at position 325 that was found to be mutated in the case described. Each color corresponds to a different percentage of amino acid conservation in different species. (**b**) Polyphen report for p.D109N mutation indicates that the mutation could be highly damaging with a score of 1.

**Figure 5:**
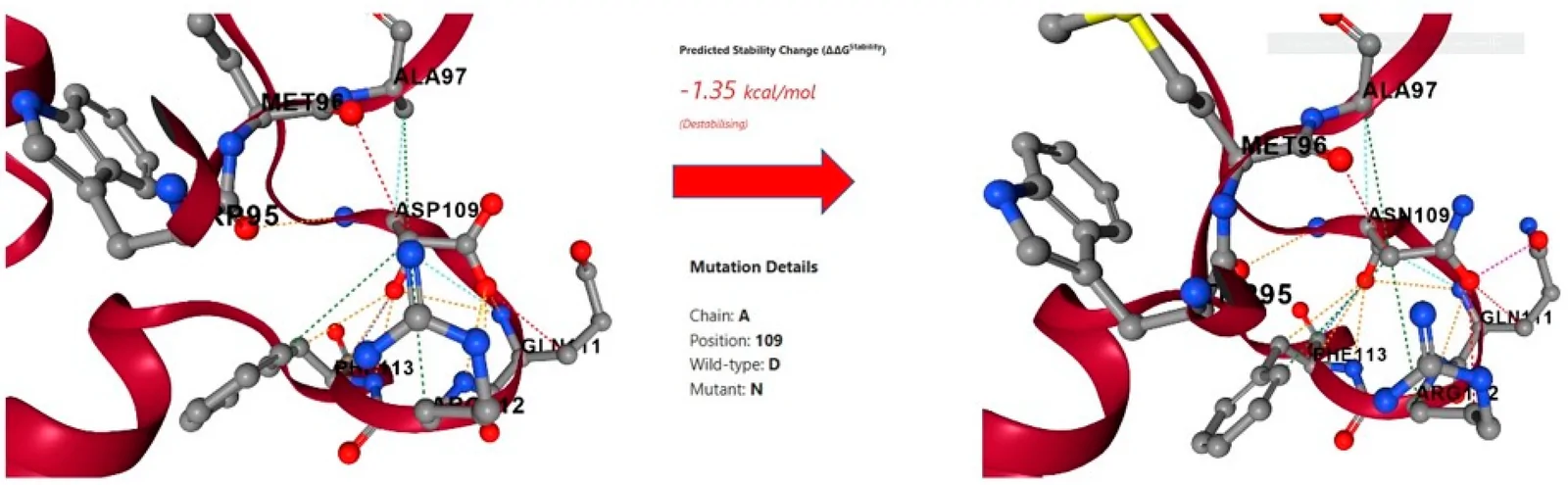
Structural analysis and stability prediction of the p.D109N variant using DynaMut2. On the left is a 3D representation of the protein, showing the Asp109 residue and the local interactions (dashed lines) involved in the network of non-covalent bonds. The Asp → Asn substitution leads to a reorganization of intermolecular interactions in the surrounding structural microenvironment. Shown on the right are the stability prediction results obtained using DynaMut2, which indicate a change in the free energy of stability (ΔΔG = −1.35 kcal/mol), consistent with a destabilizing effect of the mutation on the protein structure compared with the wild-type form.

**Figure 6:**
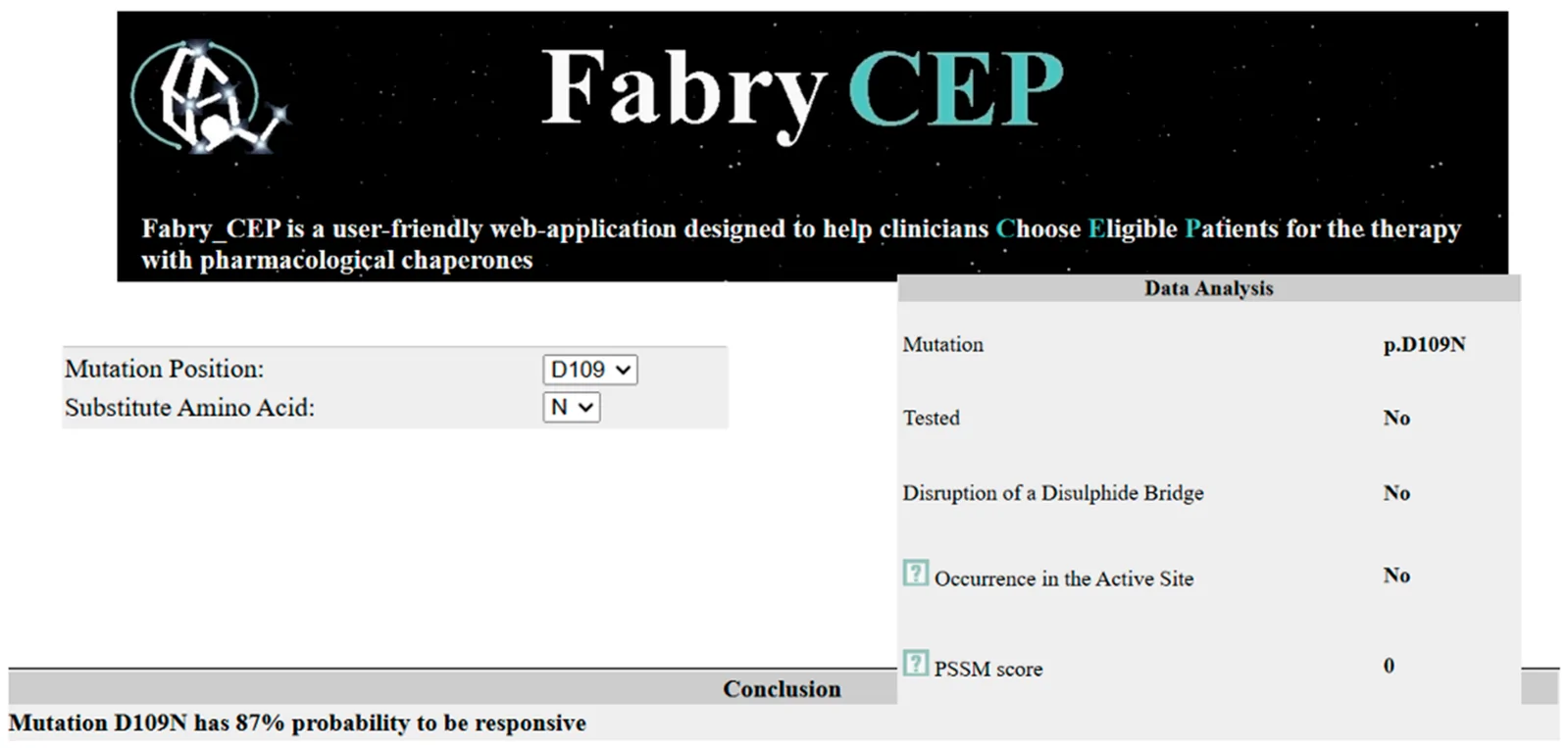
The Fabry_CEP report for the p.D109N mutation specifies the characteristics of the mutation and whether patients are eligible for treatment with pharmacological chaperones. The two question marks (?) next to “Occurrence in the Active Site” and “PSSM score” are help/information icons; they are not part of the result.

**Figure 7:**
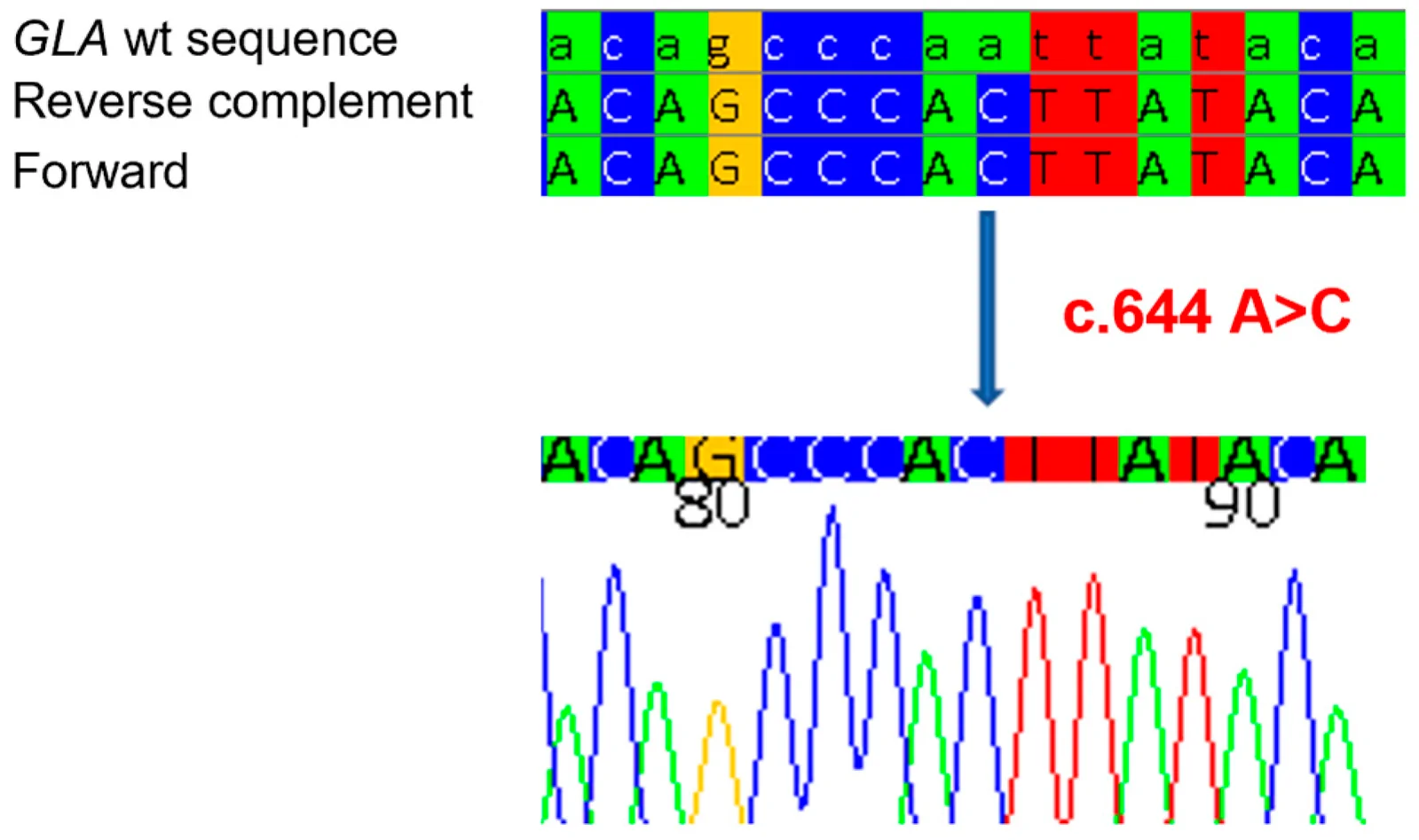
Sanger sequencing shows the c.644 A>C mutation in hemizygous state in the proband. Each base corresponds to a color: red for Thymine, blue for Cytosine, yellow for Guanine, green for Adenine. Arrows indicate the mutation.

**Figure 8:**
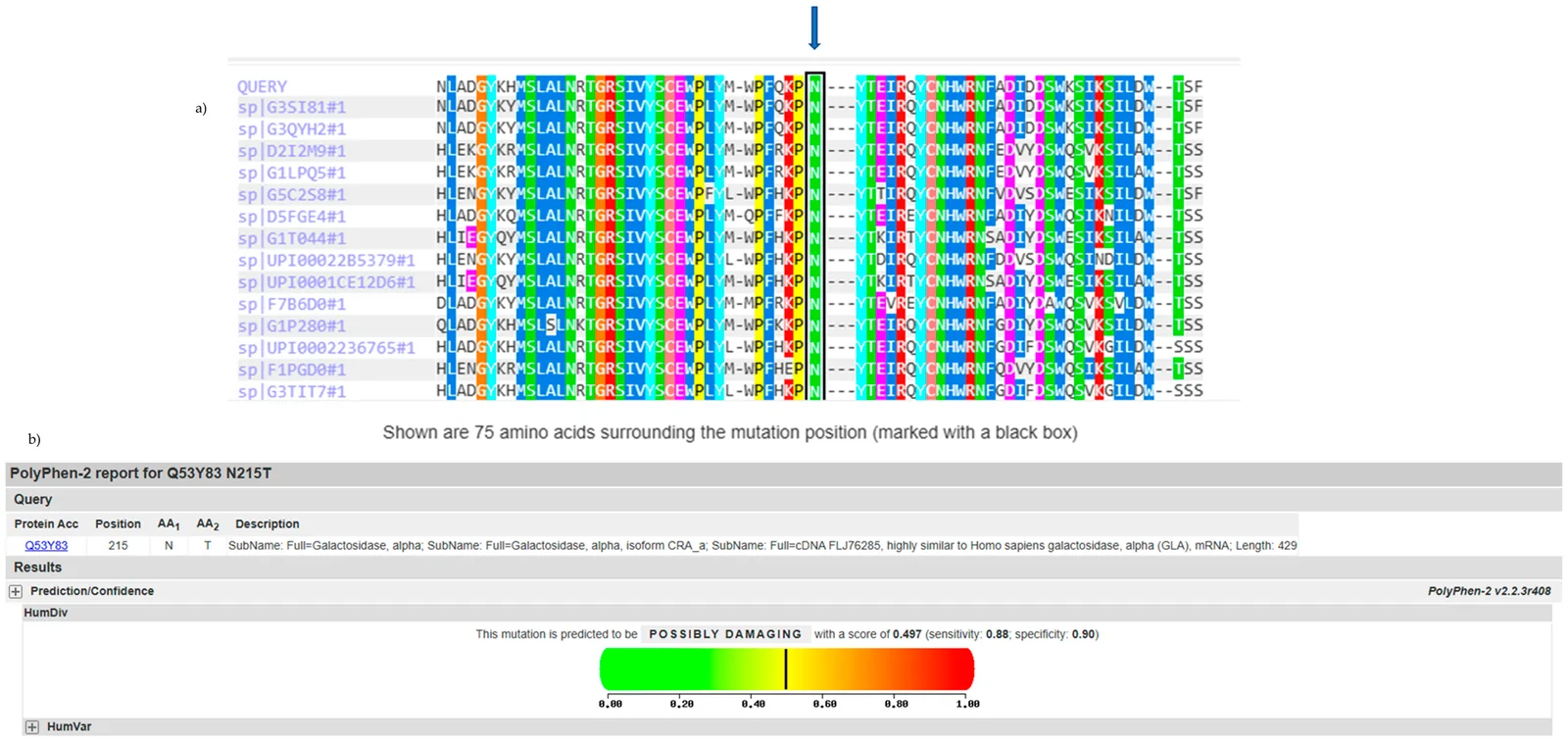
(**a**) Alignment of human α-Gal A amino acids with sequences from other organisms. The arrow indicates the amino acid at position 644 that was found to be mutated in the case described. Each color corresponds to a different percentage of amino acid conservation in different species. (**b**) Polyphen report for N215T mutation indicates that the mutation could be highly damaging with a score of 0.497.

**Figure 9:**
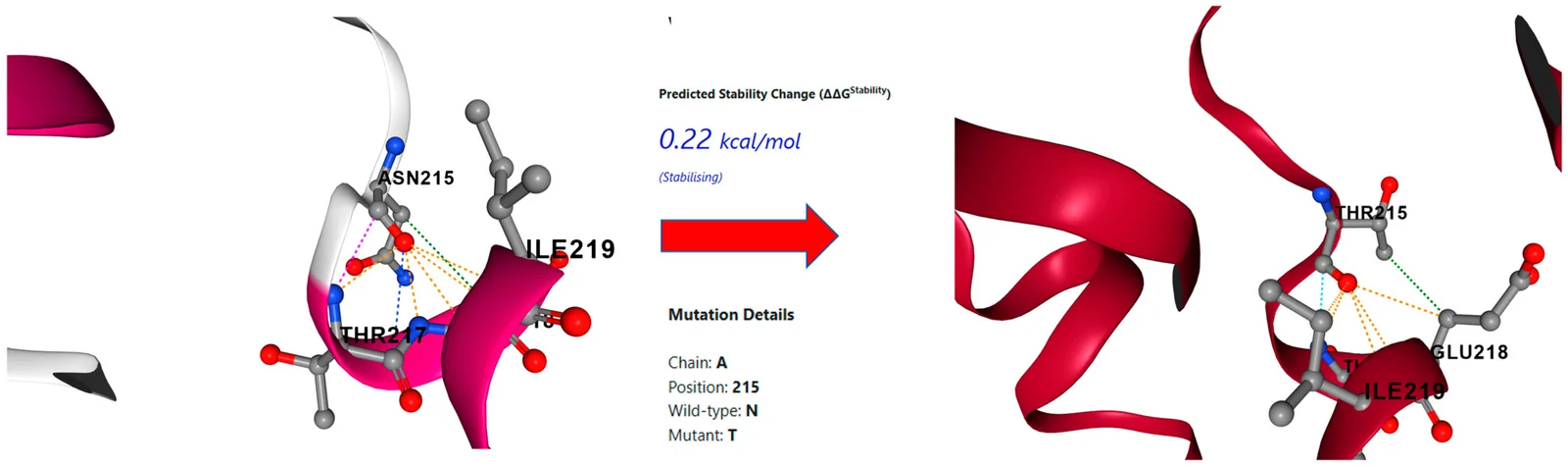
Structural analysis and stability prediction of the p.N215T variant using DynaMut2. The 3D representation of the protein is shown on the left, highlighting the Asn215 residue and the local interactions (dashed lines) involved in the network of non-covalent bonds. The Asn → Thr substitution leads to a reorganization of intermolecular interactions in the surrounding structural microenvironment. On the right are the stability prediction results obtained using DynaMut2, which indicate a change in the free energy of stability (ΔΔG = 0.22 kcal/mol), consistent with a relatively insignificant effect of the mutation on protein stability compared with the wild-type form.

**Figure 10:**
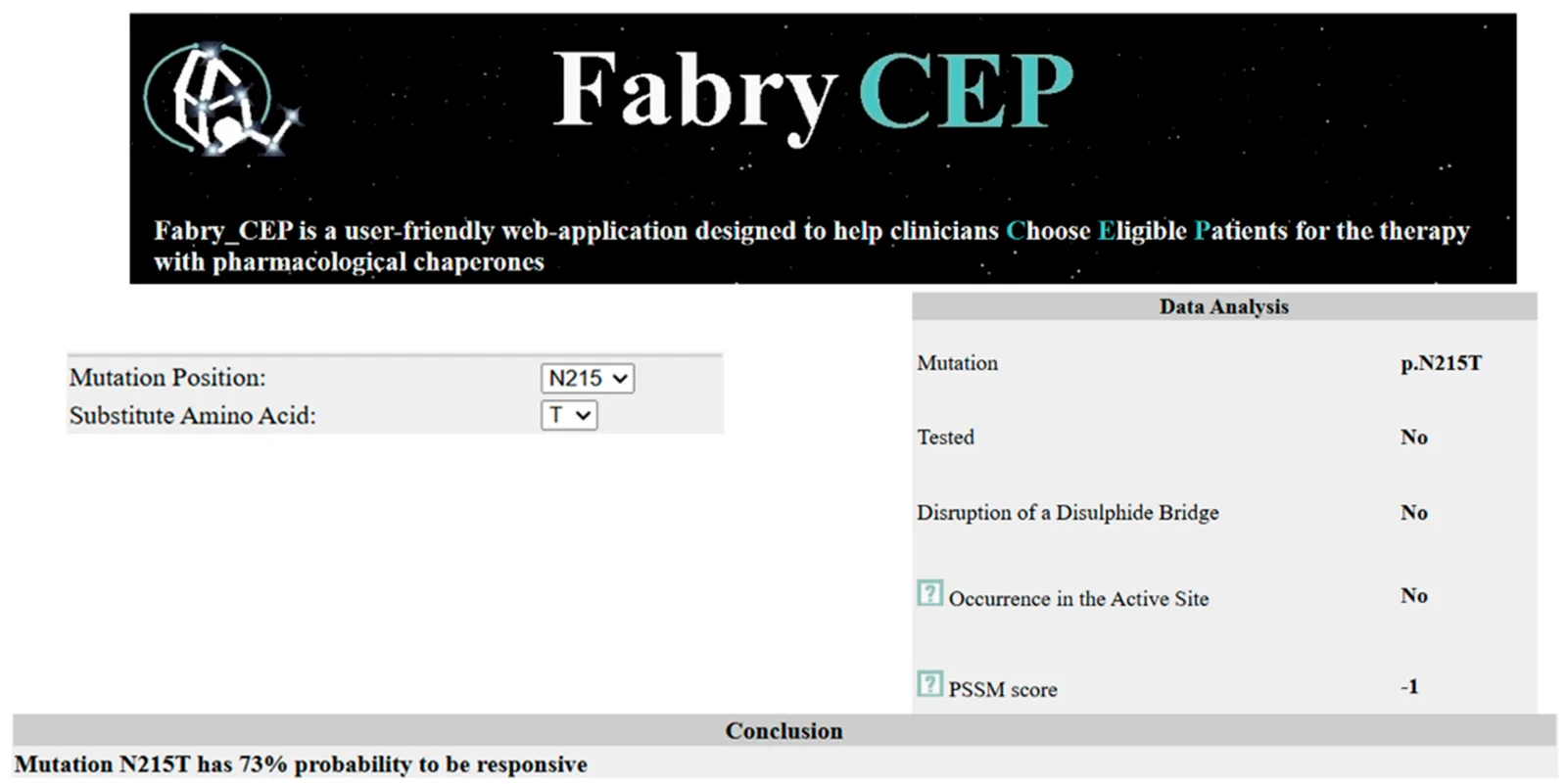
The Fabry_CEP report for the p.N215T mutation specifies the characteristics of the mutation and whether patients are eligible for treatment with pharmacological chaperones. The two question marks (?) next to “Occurrence in the Active Site” and “PSSM score” are help/information icons; they are not part of the result.

**Figure 11:**
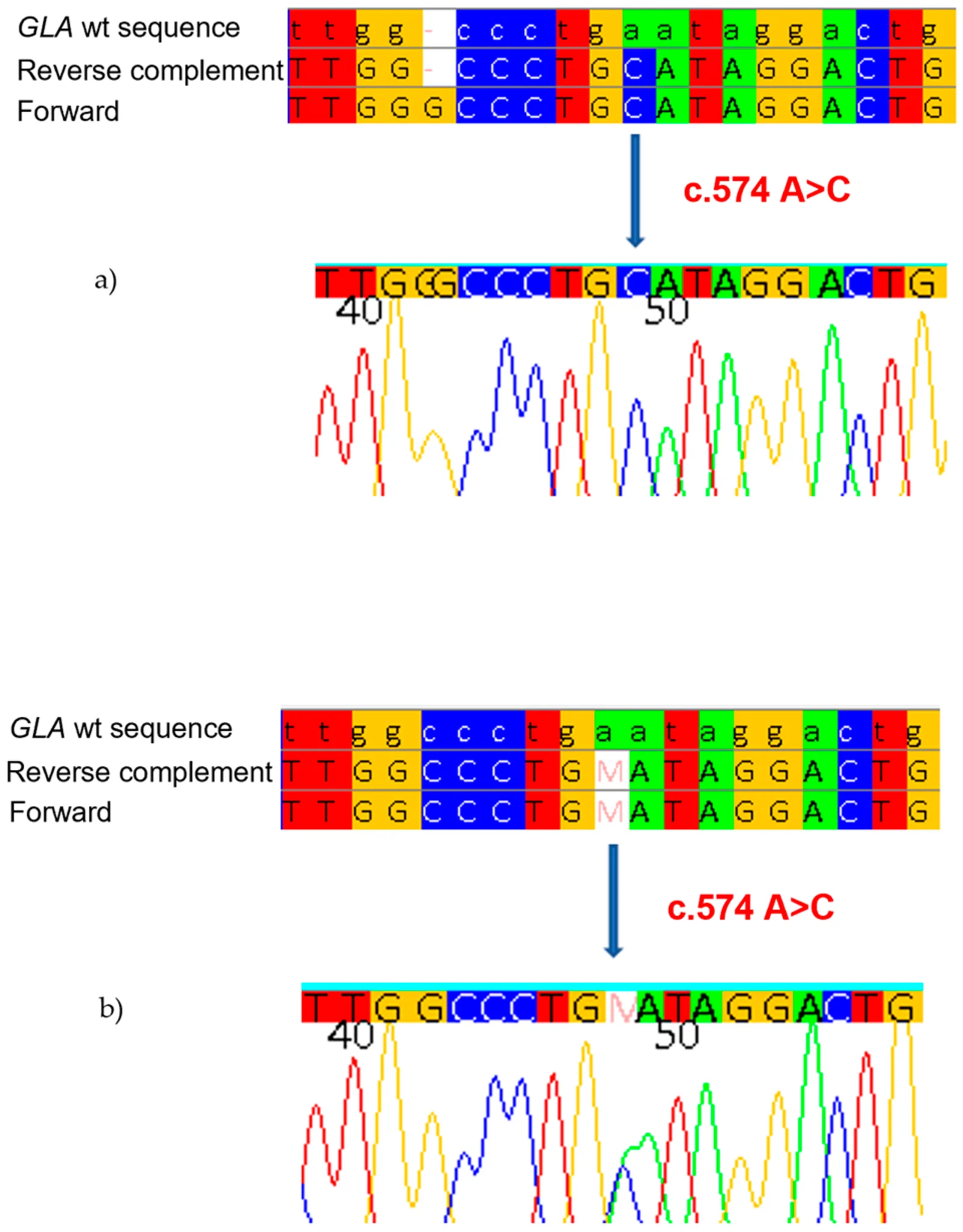
(**a**) Sanger sequencing shows the c.574 A>C mutation in hemizygous state in the proband and (**b**) in heterozygous state in the proband’s daughter. The pink letter “M” indicates an ambiguous nucleotide position (A/C) according to IUPAC nomenclature, consistent with the presence of the c.574A>C variant in a heterozygous state. Each base corresponds to a color: red for Thymine, blue for Cytosine, yellow for Guanine, green for Adenine. Arrows indicate the mutation.

**Figure 12:**
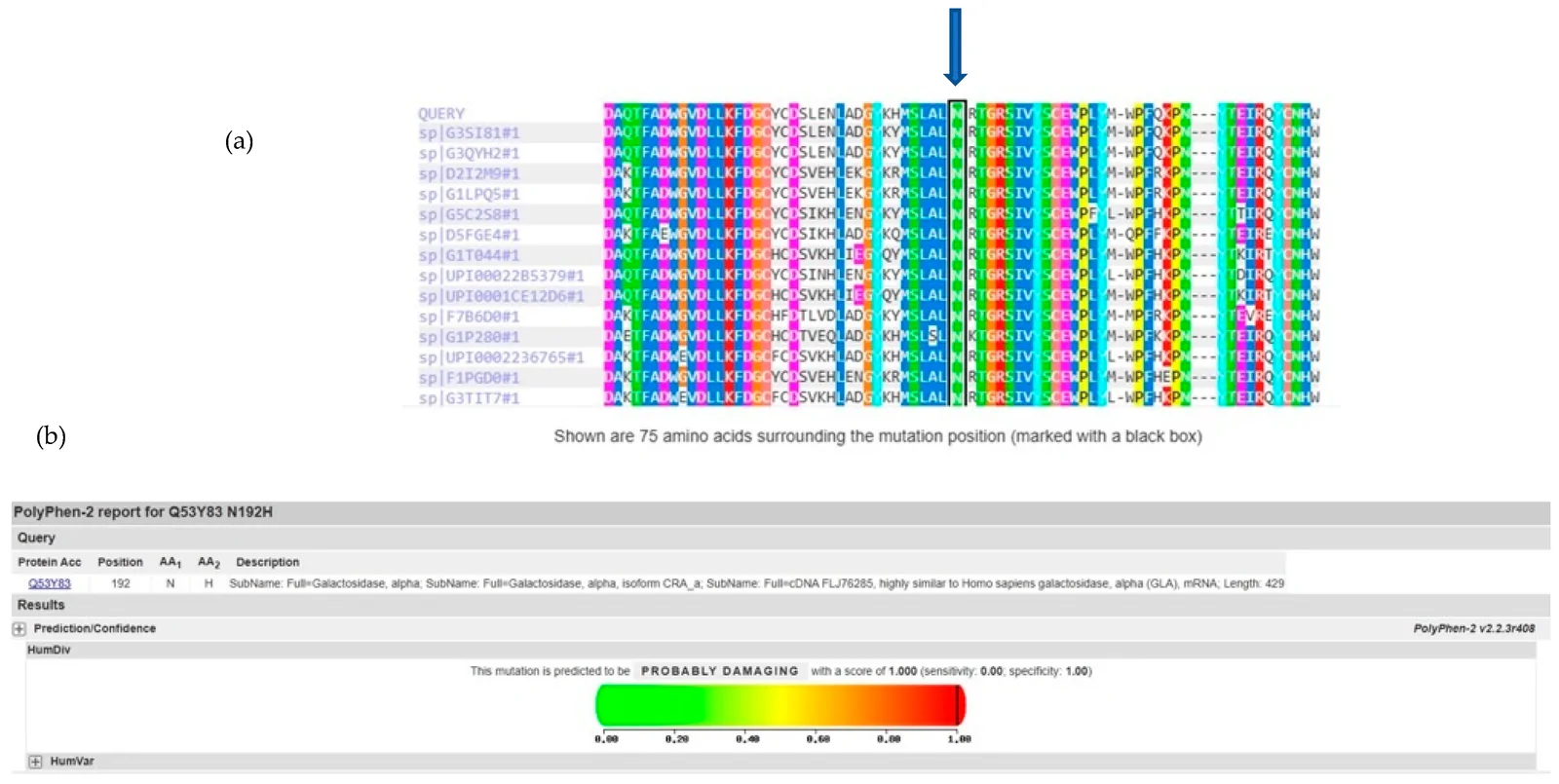
(**a**) Alignment of human α-Gal A amino acids with sequences from other organisms. The arrow indicates the amino acid at position 574 that was found to be mutated in the case described. Each color corresponds to a different percentage of amino acid conservation in different species. (**b**) Polyphen report for the N192H mutation indicates that the mutation could be highly damaging with a score of 1.

**Figure 13:**
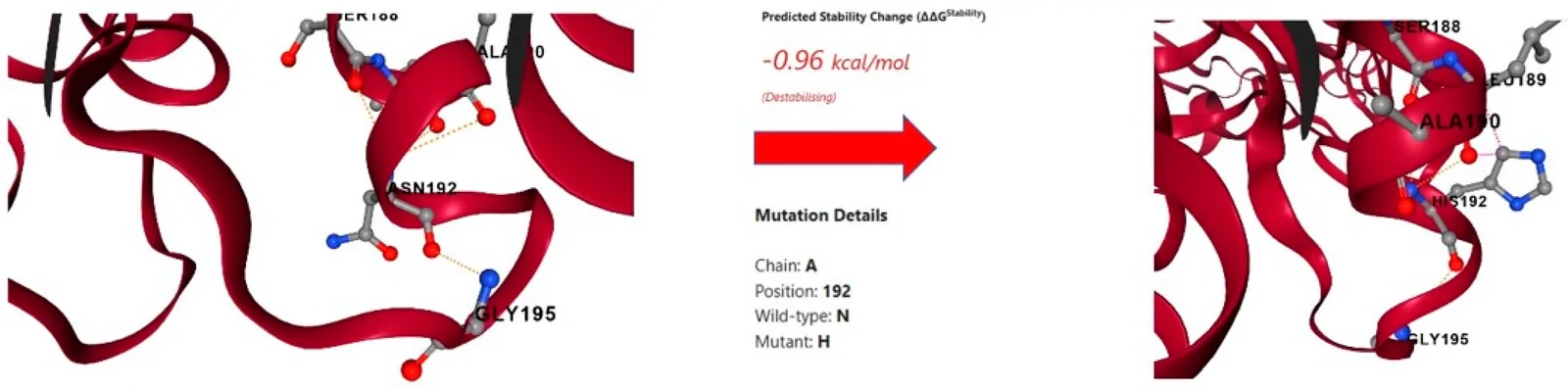
Structural analysis and stability prediction of the p.N192H variant using DynaMut2. The 3D representation of the protein is shown on the left, highlighting the Asn192 residue and the local interactions (dashed lines) involved in the network of non-covalent bonds. The Asn → His substitution leads to a reorganization of intermolecular interactions in the surrounding structural microenvironment. On the right are the stability prediction results obtained using DynaMut2, which indicate a change in the free energy of stability (ΔΔG = 0.96 kcal/mol), consistent with a destabilizing effect of the mutation on the protein structure compared with the wild-type form.

**Figure 14:**
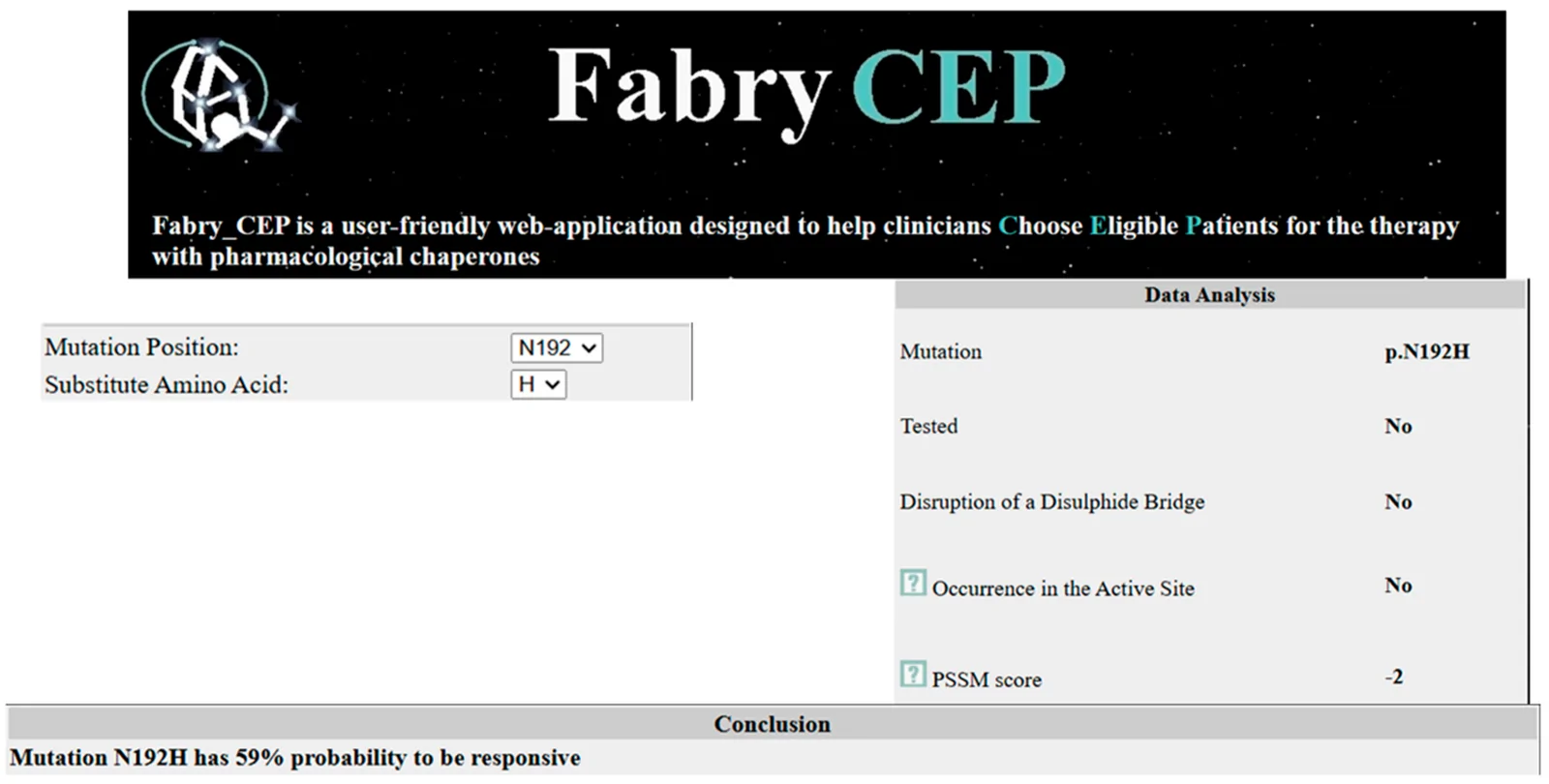
The Fabry_CEP report for the p.N192H mutation specifies the characteristics of the mutation and whether patients are eligible for treatment with pharmacological chaperones. The two question marks (?) next to “Occurrence in the Active Site” and “PSSM score” are help/information icons; they are not part of the result.

**Figure 15:**
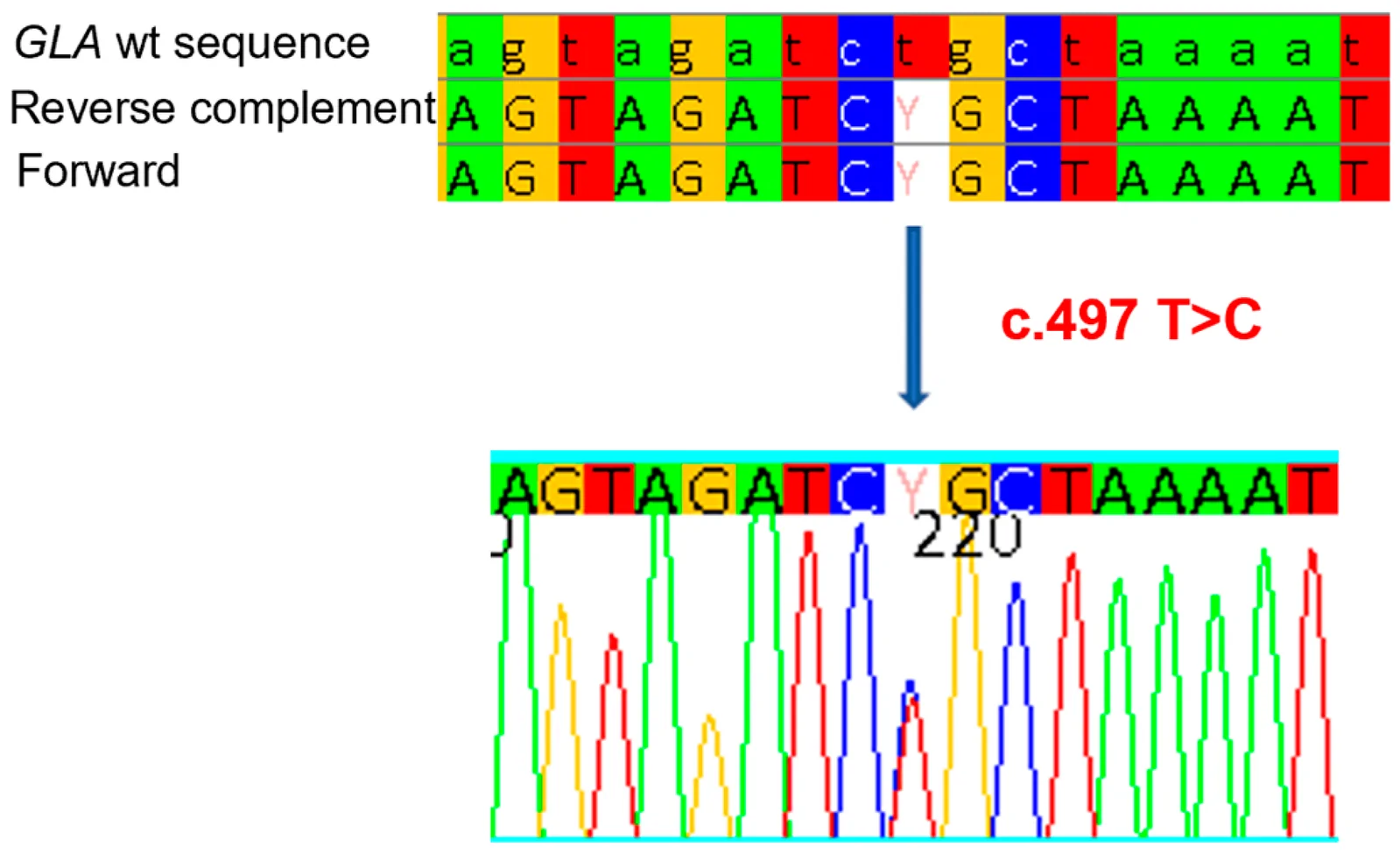
Sanger sequencing shows the c.497 T>C mutation in heterozygosity in the proband. The pink letter “Y” indicates an ambiguous nucleotide position (C/T) according to IUPAC nomenclature, consistent with the presence of the c.497 T>C variant in a heterozygous state. Each base corresponds to a color: red for Thymine, blue for Cytosine, yellow for Guanine, green for Adenine. Arrows indicate the mutation.

**Figure 16:**
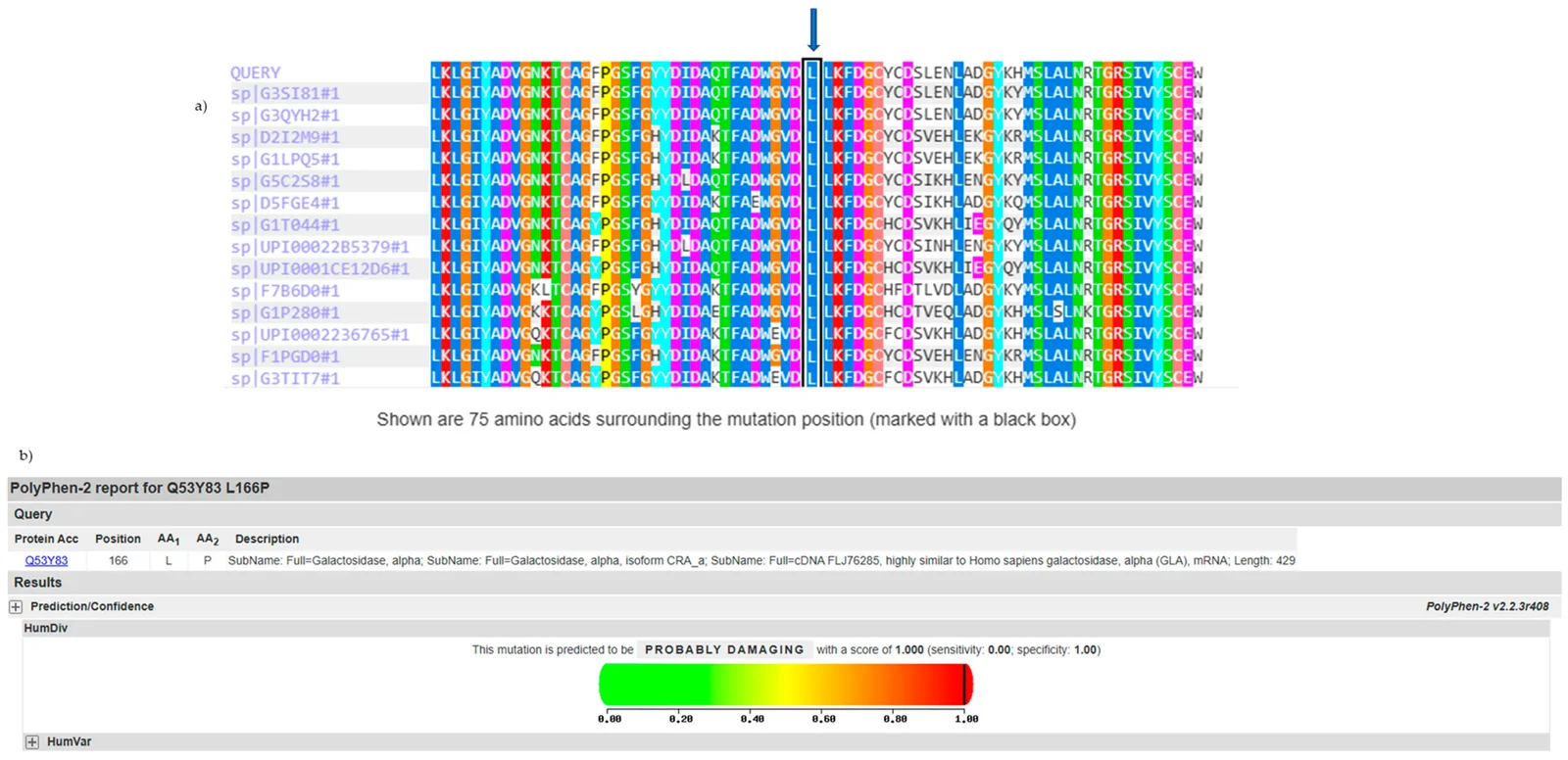
(**a**) Alignment of human α-Gal A amino acids with sequences from other organisms. The arrow indicates the amino acid at position 497 that was found to be mutated in the case described. Each color corresponds to a different percentage of amino acid conservation in different species. (**b**) Polyphen report for L166P mutation indicates that the mutation could be highly damaging with a score of 1.

**Figure 17:**
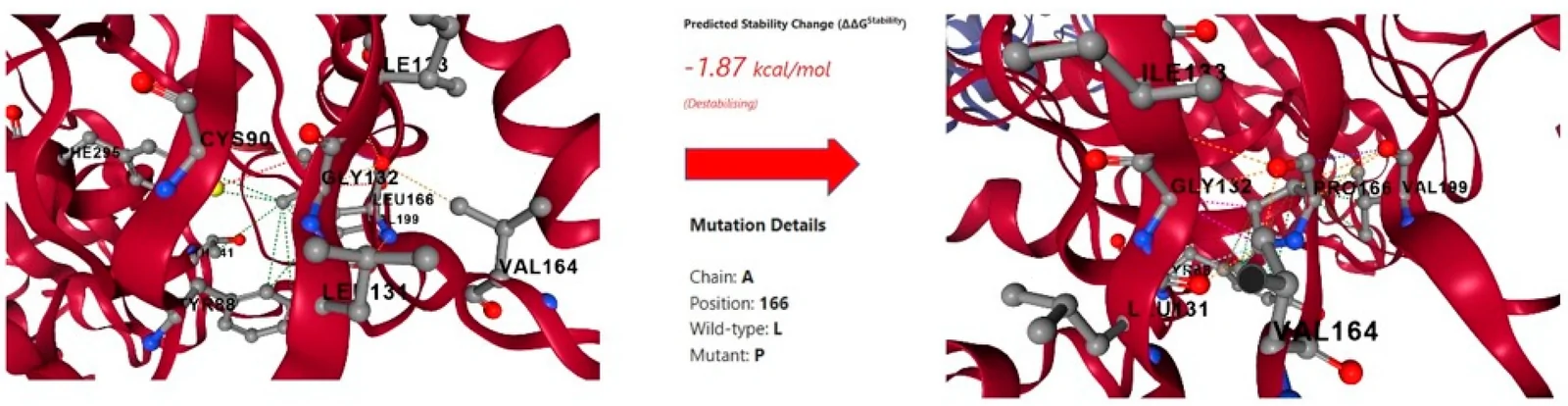
Structural analysis and stability prediction of the p.L166P variant using DynaMut2. The 3D representation of the protein is shown on the left, highlighting the Leu166 residue and the local interactions (dashed lines) involved in the network of non-covalent bonds. The Leu → Pro substitution leads to a reorganization of intermolecular interactions within the surrounding structural microenvironment. On the right are the stability prediction results obtained using DynaMut2, which indicate a change in the free energy of stability (ΔΔG = −1.87 kcal/mol), consistent with a destabilizing effect of the mutation on the protein structure compared with the wild-type form.

**Figure 18:**
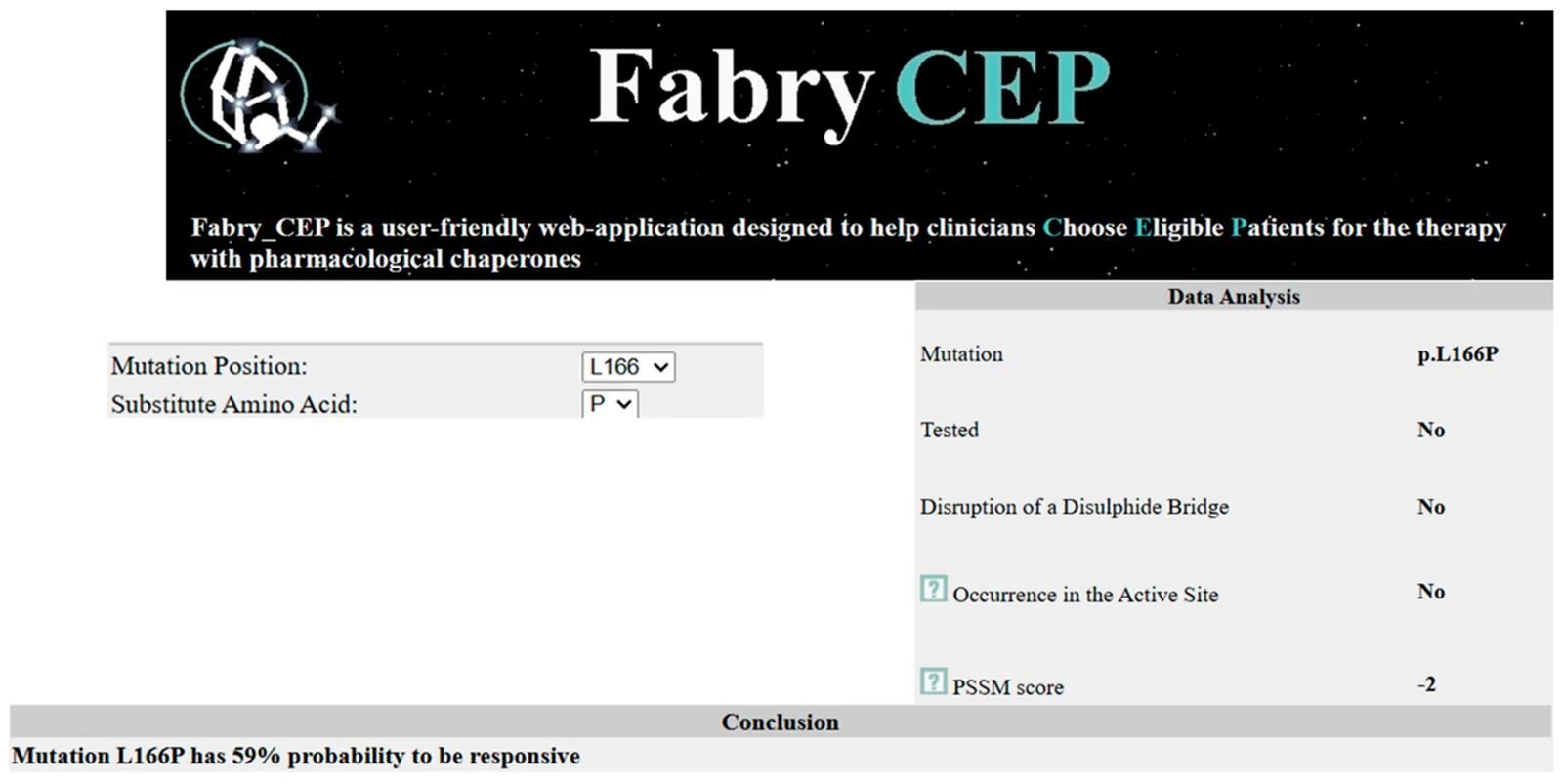
The Fabry_CEP report for the p.L166P mutation specifies the characteristics of the mutation and whether patients are eligible for treatment with pharmacological chaperones. The two question marks (?) next to “Occurrence in the Active Site” and “PSSM score” are help/information icons; they are not part of the result.

**Figure 19:**
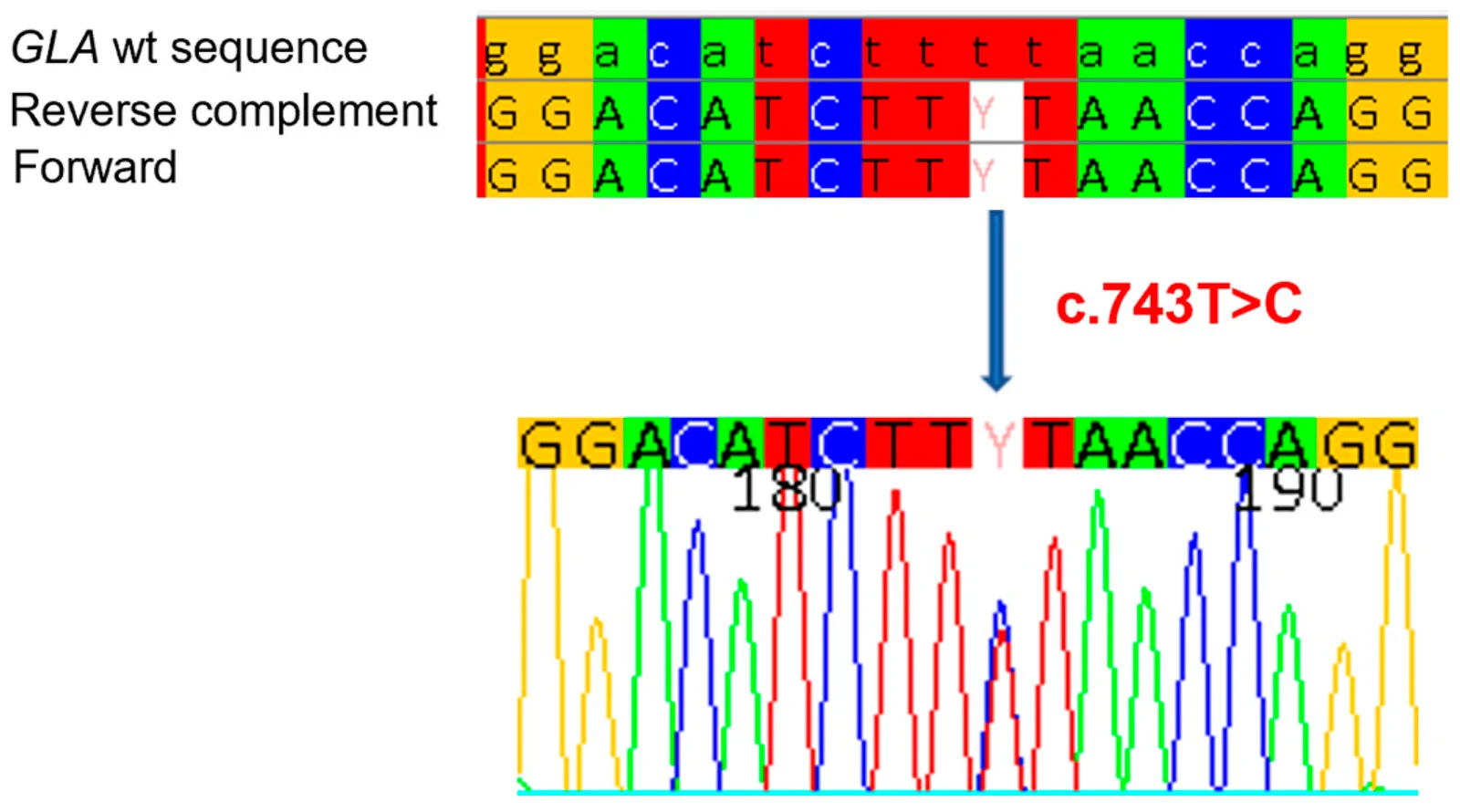
Sanger sequencing shows the c.743 T>C mutation in heterozygosity in the proband. The pink letter “Y” indicates an ambiguous nucleotide position (C/T) according to IUPAC nomenclature, consistent with the presence of the c.743 T>C variant in a heterozygous state. Each base corresponds to a color: red for Thymine, blue for Cytosine, yellow for Guanine, green for Adenine. Arrows indicate the mutation.

**Figure 20:**
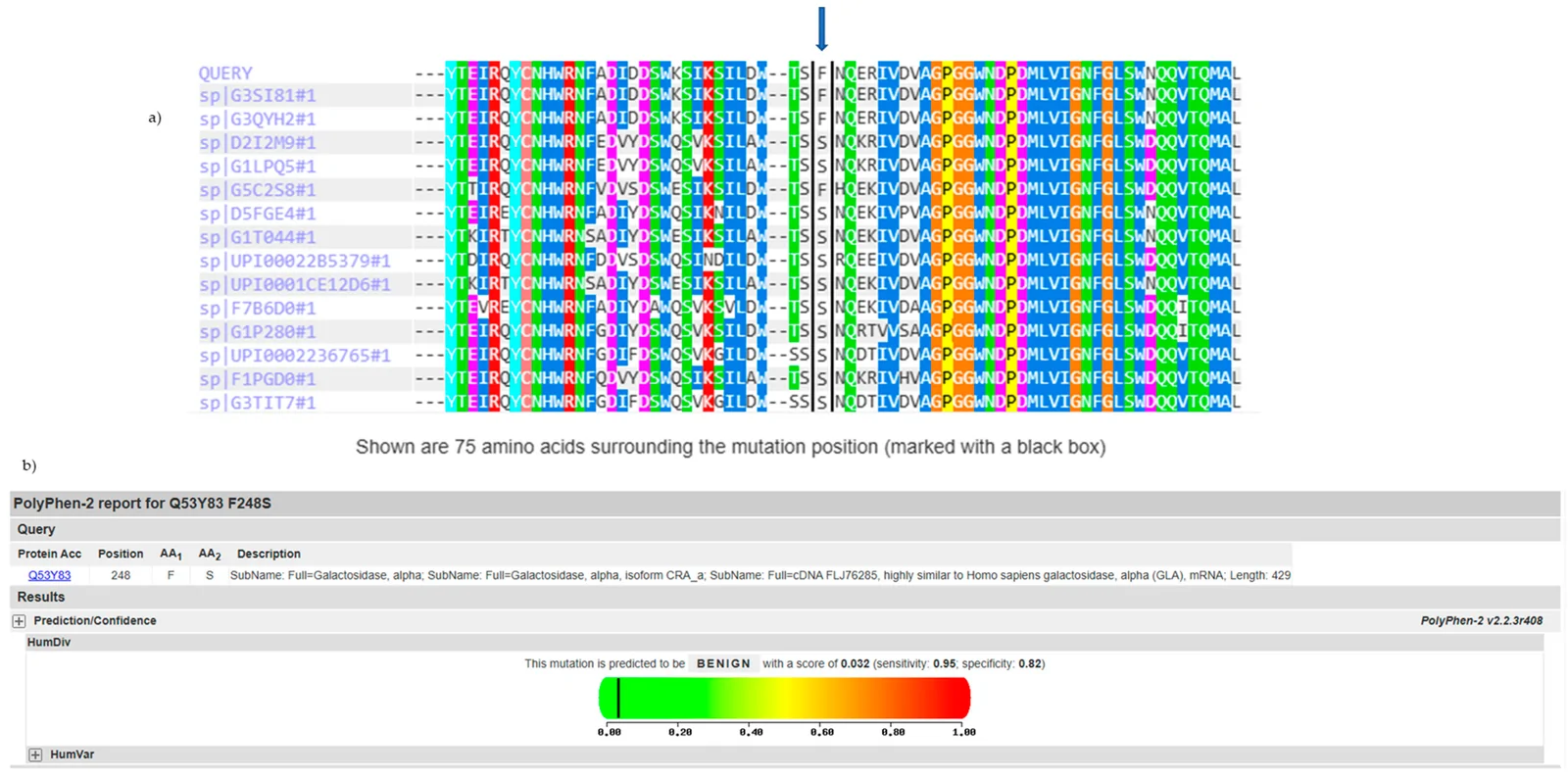
(**a**) Alignment of human α-Gal A amino acids with sequences from other organisms. The arrow indicates the amino acid at position 743 that was found to be mutated in the case described. Each color corresponds to a different percentage of amino acid conservation in different species. (**b**) Polyphen report for F248S mutation indicates that the mutation could be benign with a score of 0.032.

**Figure 21:**
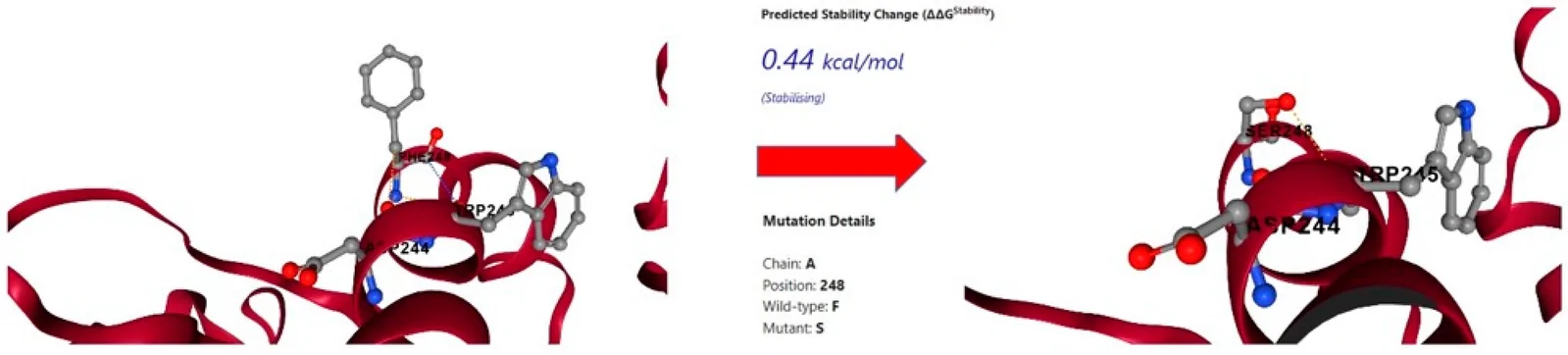
Structural analysis and stability prediction of the p.F248S variant using DynaMut2. On the left is a 3D representation of the protein, highlighting the Phe248 residue and the local interactions (dashed lines) involved in the network of non-covalent bonds. The Phe → Ser substitution leads to a reorganization of the intermolecular interactions in the surrounding structural microenvironment. On the right are the stability prediction results obtained using DynaMut2, which indicate a change in the free energy of stability (ΔΔG = 0.44 kcal/mol), consistent with a slight destabilizing effect of the mutation on the protein structure compared with the wild-type form, without altering the enzyme’s functionality.

**Figure 22:**
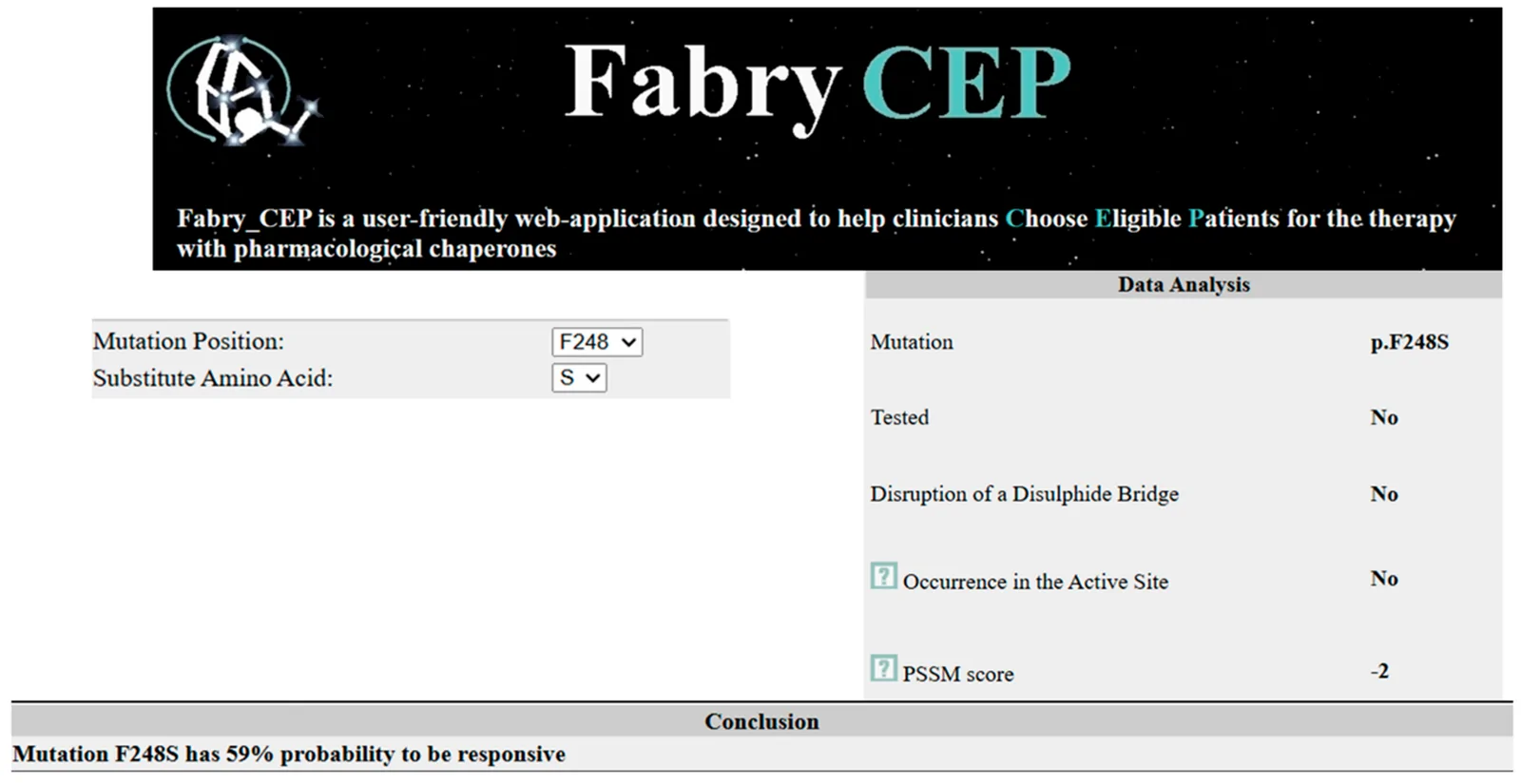
The Fabry_CEP report for the p.F248S mutation specifies the characteristics of the mutation and whether patients are eligible for treatment with pharmacological chaperones. The two question marks (?) next to “Occurrence in the Active Site” and “PSSM score” are help/information icons; they are not part of the result.

**Table 1:** Molecular and clinical data of patients with the p.D109N mutation. Bold indicates pathological values.

Patient	Sex	Age	Mutation	Alpha-Galactosidase A Activity (nmol/mL/h) Normal Range: >3.0 nmol/mL/h	Lyso-Gb3 in Blood (nmol/L) Normal Range: <2.3 nmol/L	Clinical Signs
Proband	M	5 months	p.D109N	**0.5**	1.78	No info

**Table 2:** Molecular and clinical data of the patient with the p.N215T mutation (LVH: left ventricular hypertrophy). Bold indicates pathological values.

Patient	Sex	Age	Mutation	Alpha-Galactosidase A Activity (nmol/mL/h) Normal Range: >3.0 nmol/mL/h	Lyso-Gb3 in Blood (nmol/L) Normal Range: <2.3 nmol/L	Clinical Signs
Proband	M	56	p.N215T	**0.8**	**21.38**	LVH, proteinuria

**Table 3:** Molecular and clinical data of patients with the p.N192H mutation (LVH: left ventricular hypertrophy). Bold indicates pathological values.

Patient	Sex	Age	Mutation	Alpha-Galactosidase A Activity (nmol/mL/h) Normal Range: >3.0 nmol/mL/h	Lyso-Gb3 in Blood (nmol/L) Normal Range: <2.3 nmol/L	Clinical Signs
Proband	M	56	p.N192H	**1.1**	**29.14**	LVH, dialysis
Daughter	F	26	p.N192H	9.5	**4.45**	No info

**Table 4:** Molecular and clinical data of the patient with the p.L166P mutation (LVH: left ventricular hypertrophy). Bold indicates pathological values.

Patient	Sex	Age	Mutation	Alpha-Galactosidase A Activity (nmol/mL/h) Normal Range: >3.0 nmol/mL/h	Lyso-Gb3 in Blood (nmol/L) Normal Range: <2.3 nmol/L	Clinical Signs
Proband	F	57	p.L166P	3.5	**17.05**	LVH

**Table 5:** Molecular and clinical data of the patient with the p.F248S mutation (TIA: transient ischemic attack).

Patient	Sex	Age	Mutation	Alpha-Galactosidase A Activity (nmol/mL/h) Normal Range: >3.0 nmol/mL/h	Lyso-Gb3 in Blood (nmol/L) Normal Range: <2.3 nmol/L	Clinical Signs
Proband	F	48	p.F248S	16.2	1.53	Acroparesthesia, TIA or ictus

**Table 6:** ACMG classifications of the five new variants described in this article.

Mutation	Criteria for Classifying Pathogenic Variants	ACMG Classification
D109N	PM1, PM2, PS3, PP3	Likely Pathogenic
N215T	PM1, PM2, PM5, PP3	Likely Pathogenic
N192H	PM1, PM2, PP1, PP3	Likely Pathogenic
L166P	PM1, PM2, PS3, PP3	Likely Pathogenic
F248S	PM2	VUS

## Data Availability

The original contributions presented in this study are included in the article. Further inquiries can be directed to the corresponding author.
